# Glucose sensor-augmented continuous subcutaneous insulin infusion in patients with diabetic gastroparesis: An open-label pilot prospective study

**DOI:** 10.1371/journal.pone.0194759

**Published:** 2018-04-13

**Authors:** Jorge Calles-Escandón, Kenneth L. Koch, William L. Hasler, Mark L. Van Natta, Pankaj J. Pasricha, James Tonascia, Henry P. Parkman, Frank Hamilton, William H. Herman, Marina Basina, Bruce Buckingham, Karen Earle, Kjersti Kirkeby, Kristen Hairston, Tamis Bright, Amy E. Rothberg, Andrew T. Kraftson, Elias S. Siraj, Angela Subauste, Linda A. Lee, Thomas L. Abell, Richard W. McCallum, Irene Sarosiek, Linda Nguyen, Ronnie Fass, William J. Snape, Ivana A. Vaughn, Laura A. Miriel, Gianrico Farrugia, the NIDDK Gastroparesis Clinical Research Consortium (GpCRC)

**Affiliations:** 1 Endocrinology Section, MetroHealth Regional, Case Western Reserve University, Cleveland, Ohio, United States of America; 2 Section on Gastroenterology, Wake Forest University, Winston-Salem, North Carolina, United States of America; 3 Division of Gastroenterology, University of Michigan, Ann Arbor, Michigan, United States of America; 4 Departments of Biostatistics and Epidemiology, Johns Hopkins University Bloomberg School of Public Health, Baltimore, Maryland, United States of America; 5 Section of Gastroenterology, Johns Hopkins School of Medicine, Baltimore, Maryland, United States of America; 6 Section of Gastroenterology, Temple University School of Medicine, Philadelphia, Pennsylvania, United States of America; 7 National Institute of Diabetes and Digestive and Kidney Diseases, Bethesda, Maryland, United States of America; 8 Division of Metabolism, Endocrinology, and Diabetes, University of Michigan, Ann Arbor, Michigan, United States of America; 9 Division of Pediatric Endocrinology, Stanford University School of Medicine, Palo Alto, California, United States of America; 10 Division of Endocrinology, California Pacific Medical Center, San Francisco, California, United States of America; 11 Section of Endocrinology, Wake Forest University, Winston-Salem, North Carolina, United States of America; 12 Division of Endocrinology, Diabetes, and Metabolism, Texas Tech University School of Medicine, El Paso, Texas, United States of America; 13 Section of Endocrinology, Temple University School of Medicine, Philadelphia, Pennsylvania, United States of America; 14 Division of Endocrinology, University of Mississippi, Jackson, Mississippi, United States of America; 15 Division of Gastroenterology, University of Louisville School of Medicine, Louisville, Kentucky, United States of America; 16 Section of Gastroenterology, Texas Tech University School of Medicine, El Paso, Texas, United States of America; 17 Division of Gastroenterology, Stanford University School of Medicine, Palo Alto, California, United States of America; 18 Gastroenterology Division, MetroHealth Regional, Case Western Reserve University, Cleveland, Ohio, United States of America; 19 Division of Gastroenterology, California Pacific Medical Center, San Francisco, California, United States of America; 20 Section of Gastroenterology, Mayo Clinic, Rochester, Minnesota, United States of America; Newcastle University Institute of Cellular Medicine, UNITED KINGDOM

## Abstract

Erratic blood glucose levels can be a cause and consequence of delayed gastric emptying in patients with diabetes. It is unknown if better glycemic control increases risks of hypoglycemia or improves hemoglobin A1c levels and gastrointestinal symptoms in diabetic gastroparesis. This study investigated the safety and potential efficacy of continuous subcutaneous insulin infusion (CSII) and continuous glucose monitoring (CGM) in poorly controlled diabetes with gastroparesis. Forty-five type 1 or 2 patients with diabetes and gastroparesis and hemoglobin A1c >8% from the NIDDK Gastroparesis Consortium enrolled in a 24 week open-label pilot prospective study of CSII plus CGM. The primary safety outcome was combined numbers of mild, moderate, and severe hypoglycemic events at screening and 24 weeks treatment. Secondary outcomes included glycemic excursions on CGM, hemoglobin A1c, gastroparesis symptoms, quality-of-life, and liquid meal tolerance. Combined mild, moderate, and severe hypoglycemic events occurred similarly during the screening/run-in (1.9/week) versus treatment (2.2/week) phases with a relative risk of 1.18 (95% CI 0.85–1.64, P = 0.33). CGM time in hypoglycemia (<70 mg/dL) decreased from 3.9% to 1.8% (P<0.0001), time in euglycemia (70–180 mg/dL) increased from 44.0% to 52.0% (P = 0.02), time in severe hyperglycemia (>300 mg/dL) decreased from 14.2% to 7.0% (P = 0.005), and hemoglobin A1c decreased from 9.4±1.4% to 8.3±1.3% (P = 0.001) on CSII plus CGM. Symptom scores decreased from 29.3±7.1 to 21.9±10.2 with lower nausea/vomiting, fullness/early satiety, and bloating/distention scores (P≤0.001). Quality-of-life scores improved from 2.4±1.1 to 3.1±1.1 (P<0.0001) and volumes of liquid nutrient meals tolerated increased from 420±258 to 487±312 mL (P = 0.05) at 24 weeks. In conclusion, CSII plus CGM appeared to be safe with minimal risks of hypoglycemic events and associated improvements in glycemic control, gastroparesis symptoms, quality-of-life, and meal tolerance in patients with poorly controlled diabetes and gastroparesis. This study supports the safety, feasibility, and potential benefits of improving glycemic control in diabetic gastroparesis.

## Introduction

Gastroparesis complicates type 1 (T1DM) and type 2 diabetes mellitus (T2DM) and is associated with impaired quality-of-life and significant health resource use [1, 2, 3]. Most individuals with gastroparesis secondary to diabetes show little symptom improvement over time despite aggressive management [4]. There are conflicting reports of the association between gastroparesis and glycemic control with one series observing increased symptoms correlating with elevated hemoglobin A1c and another showing no relation [5, 6]. In longitudinal studies over 12–25 years, gastric emptying delays remained stable despite hemoglobin A1c improvements [6, 7]. Poor glycemic control frequently causes hospitalization in diabetic gastroparesis [3].

The Diabetes Control and Complications Trial (DCCT) demonstrated intensive insulin therapy reduces retinopathy, neuropathy, and nephropathy in T1DM [8, 9, 10, 11]. Follow-up studies reported durable reductions in complications in those originally treated with intensive insulin regimens [8, 12, 13]. Recently, gastric emptying delays were related to high hemoglobin A1c levels in a DCCT subset followed over 27 years, suggesting that chronic poor glycemic control contributes to gastric impairment [14].

Because delayed gastric emptying leads to temporal mismatches between meal-time insulin delivery and nutrient absorption, clinicians have expressed concern about increased hypoglycemic events with intensifying glycemic control in diabetic gastroparesis. Earlier studies reported elevated hypoglycemia risks with intensive glycemic therapy in diabetes without gastroparesis [15, 16]. Furthermore, the feasibility and effectiveness of aggressive regimens in diabetic gastroparesis are uncertain. Continuous subcutaneous insulin infusion (CSII) reduced hemoglobin A1c levels by 1.8% and decreased hospitalizations in a study of T1DM patients with gastroparesis, but gastric symptoms were not assessed [17]. In another report, gastric emptying did not improve in T2DM patients despite 1.3% hemoglobin A1c reductions [18]. Rigorous characterizations of risks and benefits of intensive glycemic control with CSII plus continuous glucose monitoring (CGM) in diabetic gastroparesis have not been performed.

To address the utility of intensive glycemic control in patients with poorly controlled diabetes and gastroparesis, the National Institute of Diabetes and Digestive and Kidney Diseases (NIDDK) Gastroparesis Clinical Research Consortium (GpCRC) conducted the Pilot Study of the Safety, Feasibility, and Potential Efficacy of Continuous **GLU**cose **M**onitoring and **I**nsulin Pump **T**herapy in **D**iabetic **G**astroparesis (GLUMIT-DG). Our primary aim was to assess the safety in not increasing risks of hypoglycemia of CGM as an adjunct to finger stick glucose measurements in guiding CSII in these patients. Secondary aims included determinations if CSII plus CGM over 24 weeks is feasible to improve glycemia and is potentially effective in decreasing gastroparesis manifestations. Findings of this investigation are supportive of measures to improve glycemic control as part of the management of diabetic gastroparesis.

## Materials and methods

### Patient characteristics

**Fig 1:**
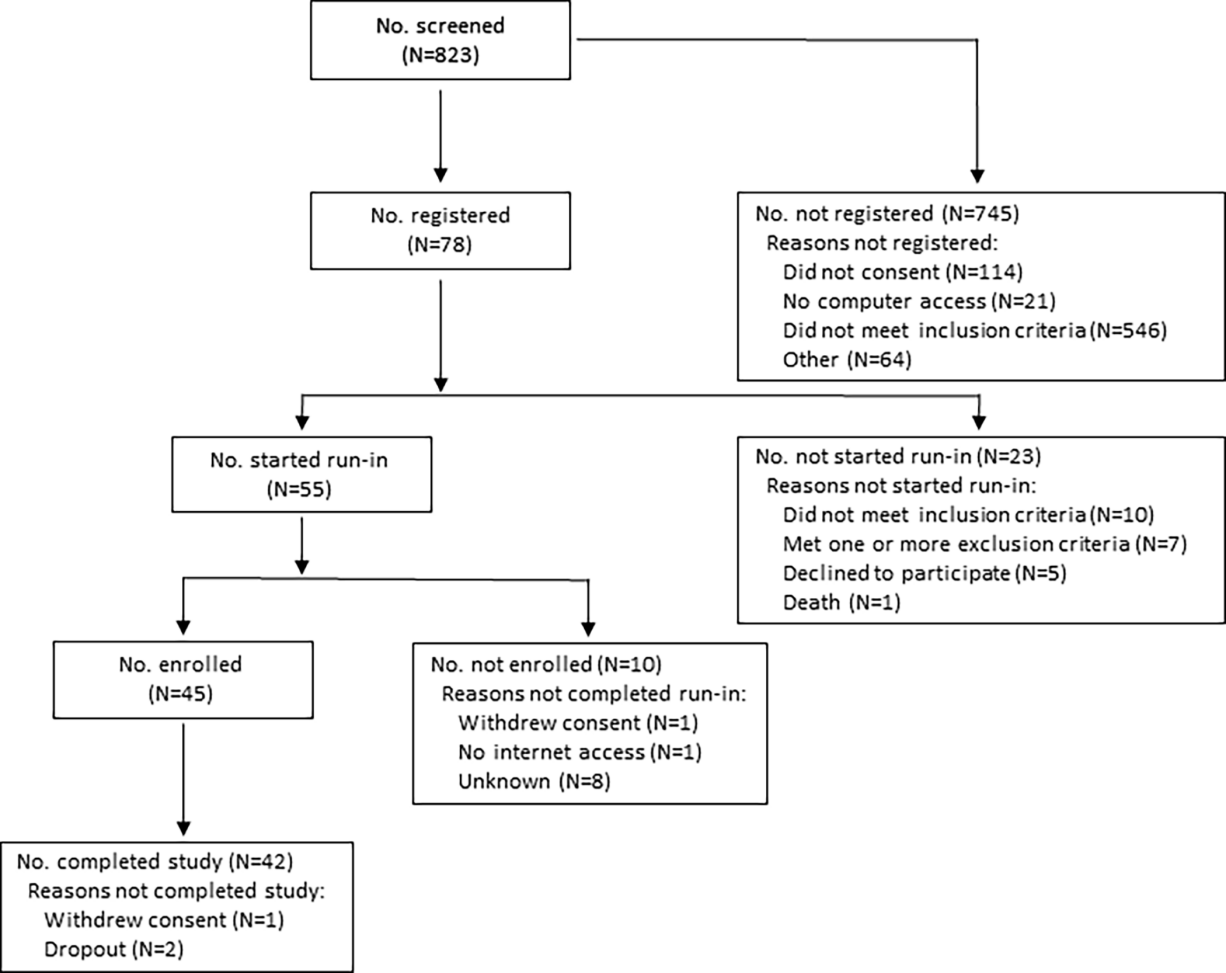
Flowchart for the GLUMIT-DG study. This figure shows the Flowchart for patient recruitment for the GLUMIT-DG study, which follows a non-randomized trial design. A TREND checklist accompanies this Flowchart as the S1 File in the Supplemental Information section.

Forty-five patients (age 18–70 years) with diabetes for ≥2 years in poor glycemic control (hemoglobin A1c >8%) and with gastroparesis were enrolled at 7 centers of the GpCRC from September 6, 2011 through April 30, 2014 [19]. The authors confirm that all ongoing and related trials for this intervention are registered (ClinicalTrials.gov Identifier NCT01030341). All week 24 follow-up visits were completed from March 5, 2012 through November 13, 2014. This clinical trial followed a non-randomized design; a patient Flowchart is provided in Fig 1. A TREND (Transparent Reporting of Evaluations with Non-randomized Designs) checklist (S1 File) is provided in the Supplemental Information section. Patients had symptoms for ≥1 year with Gastroparesis Cardinal Symptom Index (GCSI) scores ≥18 [20]. Subjects underwent upper endoscopy within one year to exclude other reasons for symptoms. Gastroparesis was confirmed with gastric scintigraphy before registration with >60% 2 hour and/or >10% 4 hour retention [19]. Determination of T1DM versus T2DM was made by investigators based on patient history and records review. Patients with diabetes already using CSII were eligible to participate. None was using CGM on enrollment. GLUMIT-DG was approved by Institutional Review Boards at each clinical center and the Data Coordinating Center as described in the Supplemental Information section (S2 File). All patients provided written informed consent. All investigations were conducted in accordance with the principles expressed in the Declaration of Helsinki. The approved initial study protocol dated August 12, 2009 (S3 File) and the approved revised protocols dated March 16, 2011 (S4 File) and April 16, 2013 (S5 File) are included in the Supplemental Information section.

### Study design

**Fig 2:**
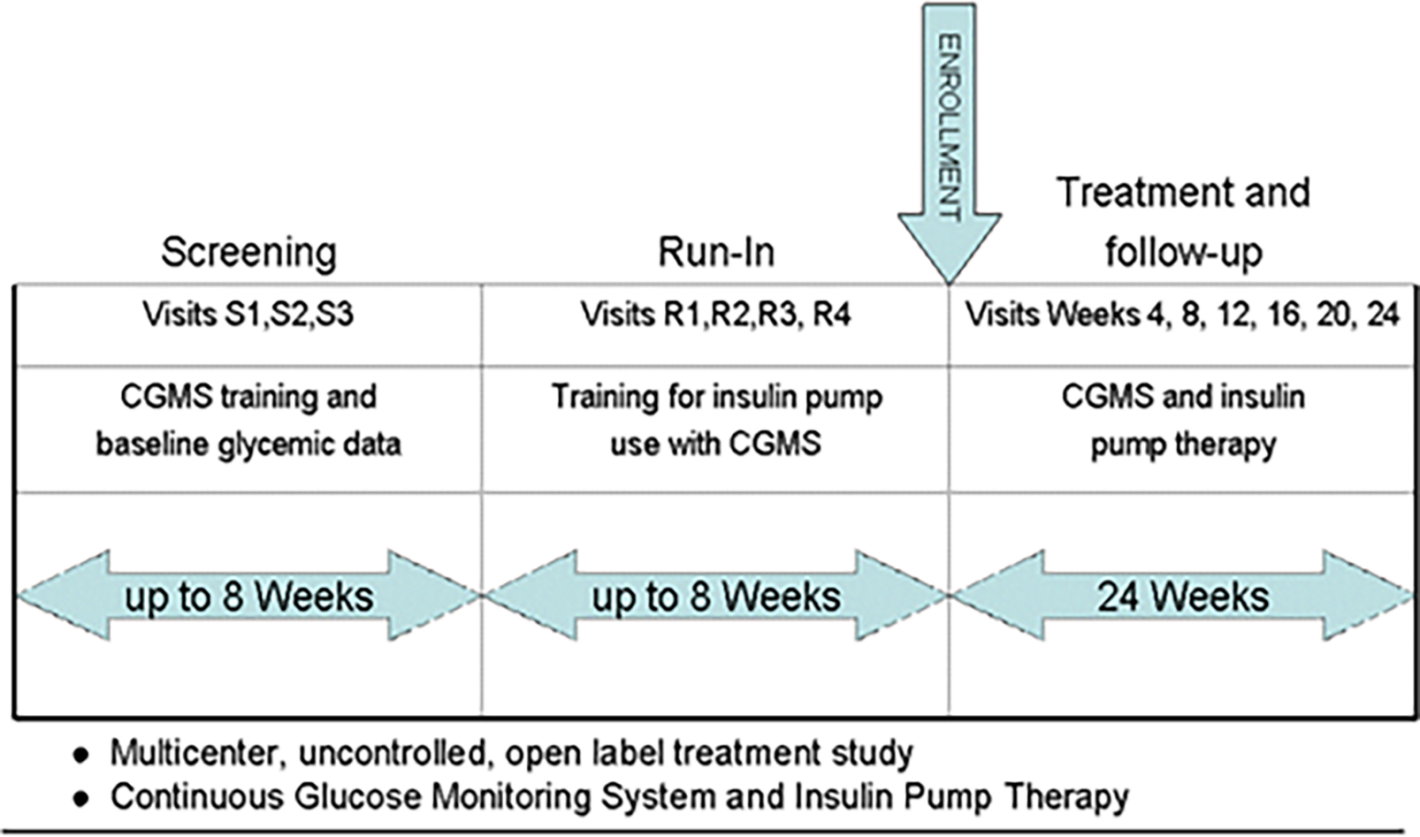
Study design for GLUMIT-DG. The study design is shown. After an initial screening phase (up to 3 visits over up to 8 weeks), a run-in phase (up to 4 visits over up to 8 weeks) was conducted. The formal treatment phase consisted of 6 study visits over 24 weeks when CSII and CGM were used together to optimize glycemic control.

GLUMIT-DG was comprised of three phases: screening, run-in, and treatment (Fig 2). Details of each phase are provided in the current study protocol in the Supplemental Information section (S5 File).

#### Screening phase (3 visits over up to 8 weeks)

Baseline glycemic profiles were obtained with blinded sensors during the screening phase (iPro2^®^ CGM, Medtronic, Northridge, CA). While wearing this device, patients did not have access to their sensor glucose values so that no insulin dosing decisions could be made based on CGM data acquired during this study phase. Patients also measured blood glucose levels 4 times daily with One Touch^®^ UltraLink or Bayer CONTOUR^®^ Next Link meters using finger stick methods. Success was defined by acquiring ≥216 hours of glycemic data over 2 weeks using both the iPro2^®^ and finger stick methods. Two additional weeks were permitted for subjects failing initial training. This study phase was designed to allow subjects to demonstrate a willingness to wear a sensor, gain proficiency in inserting a sensor into the subcutaneous tissue, and acquire and transfer baseline CGM data to the GLUMIT-DG study staff. Demographic and clinical information was acquired; diabetes therapy was not changed during screening.

#### Run-in phase (4 visits over up to 8 weeks)

Participants received detailed instructions in operating the CSII device (MiniMed Paradigm^®^ Model 722 or 723) and MiniLink^™^ REAL-Time Transmitter CGM system (Medtronic, Northridge, CA) during the run-in phase. Before enrolling in the treatment phase, subjects demonstrated competency in CSII, following glucose levels using CGM and 4 times daily finger stick testing, and electronic CGM data transfer via their home computer to GLUMIT-DG study staff. As part of the training, patients learned to adjust insulin pump infusion parameters using information collected from both finger stick glucose values and from trend analysis of CGM used as an adjunct. On run-in visit 1, CGM alarms were set. The hypoglycemic alarm threshold was set to 80 mg/dL and a low glucose snooze was set between 15–20 minutes to allow time to observe effects of treating hypoglycemia. The hyperglycemic alarm was set to 240 mg/dL with a high glucose snooze set to 1–2 hours to allow time to observe effects of additional insulin dosing. On run-in visit 2, insulin pump mechanics were taught after filling the pump with saline. Run-in visit 3 was devoted to initiating insulin therapy using CSII. Carbohydrate to insulin ratios, insulin sensitivity, insulin on board, and glycemic targets were established by study staff. In run-in visit 4, patients reviewed CGM results and received additional glycemic management education including use of CGM glucose trends as adjunctive information to finger stick glucose values to modify insulin doses. It should be noted that the threshold suspend feature was not available at the time of initiation of this project.

#### Treatment phase (visits every 4 weeks for 24 weeks)

Participants met with diabetes educators every 4 weeks during the treatment phase. During these visits, insulin pump parameters (basal rate, carbohydrate to insulin ration, etc.) were modified by patients under guidance by diabetes educators according to finger stick values and CGM glycemic trends. Stable antiemetic, prokinetic, or analgesic medication doses were permitted for symptom control.

### Continuous subcutaneous insulin infusion (CSII)

Initial basal insulin doses (T1DM—0.15 units/kg/day; T2DM—0.3 units/kg/day) were adjusted based on post-absorptive capillary glucose measurements complemented by CGM trend analyses. Meal bolus insulin recommendations included: (i) bolus initiation 15–30 minutes after eating, employing CGM to detect increasing glucose levels reflecting the onset of meal absorption, (ii) using the dual-wave feature with a small first wave (10–20% total meal dose) followed by a second wave (80–90%) over 4–6 hours, and (iii) considering temporary basal rate increases instead of meal boluses if CGM suggested longer periods of delayed meal absorption. Overall glycemic targets were 80–120 mg/dL during fasting and 100–180 mg/dL after meals.

### Primary outcome

#### Mild, moderate, and severe hypoglycemia episodes

The primary outcome comparison was the change from the screening/run-in periods to the treatment phase in weekly numbers of the sum of mild, moderate, and severe hypoglycemic episodes measured initially either on CGM or by capillary glucose and confirmed by finger stick testing at home and/or a reference blood glucose level if the patient was evaluated in a hospital setting [16]. Mild hypoglycemic episodes (50–69 mg/dL confirmed by finger stick and patient was fully capable of self-treatment) were managed with oral carbohydrates (15 grams) with retesting of blood glucose every 15–20 minutes until the glucose level exceeded 80 mg/dL. Moderate hypoglycemic episodes (<50 mg/dL confirmed by finger stick and patient was fully capable of self-treatment) were managed similarly but with larger carbohydrate amounts (20–25 grams). Severe hypoglycemic episodes (<50 mg/dL confirmed by finger stick and patient was incapable of self-treatment and required third party assistance from a friend, relative, paramedic, etc.) were managed aggressively with initial glucagon injection followed by carbohydrate ingestion (if consciousness and cooperation were restored) or additional glucagon or intravenous dextrose if indicated based on the judgment of the responsible clinician or emergency assistance personnel.

The combined rates of mild, moderate, and severe hypoglycemic episodes were chosen as the primary outcome measure because they permitted conduct of meaningful and statistically relevant analyses of the safety of combined CSII plus CGM for the proposed sample size. Using one-sample equivalence z-testing and assuming one-sided Type I error of 5%, power of 90%, 0% expected increase from screening to treatment phase, equivalence limit of 35% (0.60 vs 0.81 episodes per week), and 10% increase to allow for departures from assumptions, the calculated sample size was 40 for the primary outcome.

### Secondary outcomes

#### Serious adverse events

Severe hypoglycemic episodes were quantified separately as serious adverse events (SAEs). Other SAEs included severe hyperglycemic episodes >500 mg/dL, blood ketones ≥0.6 mmol/L, gastroparesis exacerbations (e.g. severe nausea, vomiting), altered mental status, death, life-threatening experiences, hospitalizations, prolongation of hospitalizations, emergency department attendance, and disability/incapacity. Expedited review with adjudication was required for SAEs involving severe hypoglycemic or hyperglycemic events AND any of the following: loss of consciousness, seizures, hospitalization, emergency department visit, professional intervention, or death.

Baseline factors associated with SAEs were characterized including demographic factors, medication use, screening hemoglobin A1c, gastric retention, symptom severity, quality-of-life, and meal tolerance.

#### Glycemic control

Hemoglobin A1c was measured at screening and 12 and 24 weeks treatment. CGM data acquired every 5 minutes were stratified into 2 levels of hypoglycemia (<50 and <70 mg/dL), euglycemia (70–180 mg/dL), and 2 levels of hyperglycemia (>180 and >300 mg/dL). Percent time in each glycemic range, proportions of days with ≥1 excursion in each range, and durations and weekly rates of glycemic excursions were compared during treatment versus screening. CGM readings during the run-in were not included in secondary outcome analyses.

#### Gastroparesis symptoms

The Patient Assessment of Upper Gastrointestinal Disorders Symptoms (PAGI-SYM) survey was administered at screening and 12 and 24 weeks treatment [21]. Symptom severity was quantified by the GCSI, which is comprised of 9 symptoms of the PAGI-SYM scored from 0 (no symptoms) to 5 (very severe symptoms) [20]. A total GCSI score was calculated by summing the 9 individual scores (0 to 45). Means of individual GCSI scores were calculated to provide a composite score quantifying overall symptom severity (0 to 5). GCSI nausea/vomiting, postprandial fullness/early satiety, and bloating/distention subscale scores were calculated.

#### Quality-of-life

Quality-of-life was assessed by the Patient Assessment of Upper Gastrointestinal Disorders Quality-of-Life (PAGI-QOL) survey, which scores 30 factors from 0 (none of the time) to 5 (all of the time) [22]. Mean PAGI-QOL scores averaged all factors; a score of 0 reflects poor quality-of-life. PAGI-QOL daily activities, clothing, diet and food habits, relationships, and psychological well-being domains were quantified.

#### Meal tolerance

Satiety testing measured tolerance of non-caloric and caloric liquid meals during screening and 12 and 24 weeks treatment. On the study day, patients were instructed to drink maximal volumes of water over 5 minutes until feeling completely full. Two hours later, they were instructed to drink 150 mL of Ensure^®^ (1.1 kcal/mL, Abbott Nutrition, Lake Forest, IL) every 5 minutes until feeling completely full [23]. Water and Ensure^®^ volumes ingested represented non-caloric and nutrient tolerance, respectively. Satiety tests were conducted only when fasting glucose was between 100–270 mg/dL.

### Subgroup comparisons

Post-hoc analyses were performed on T1DM and T2DM data to determine if diabetes subtype influenced primary and secondary outcomes.

### Statistical methods

Comparisons of screening characteristics between groups were assessed using Fisher’s exact test for categorical variables and independent t-tests for continuous variables. Within-patient comparisons were assessed using Poisson regression for counts and logistic regression for binary outcomes accounting for within patient correlation using generalized estimating equations with independent working correlation. Between-group comparisons of change in continuous outcomes at 12 or 24 weeks were assessed using an ANCOVA model regressing change on an indicator variable for group and baseline values of the outcome. Within-group comparisons of change in continuous outcomes were assessed using paired t-tests. P-values are nominal and two-sided. Because a goal of these pilot analyses was to generate new hypotheses to be tested in future confirmatory studies, correction for multiple comparisons was not performed. Such adjustments reduce the power of an investigation to define important differences, are unnecessary if exploratory research questions are unrelated, and are only required for studies which aim to offer decisive proof of a predefined hypothesis to endorse decision-making protocols [24, 25, 26]. Statistical analyses were performed with SAS version 9.4 (SAS Institute, Cary, NC) and Stata release 13 (Stata-Corp, College Station, TX).

## Results

### Baseline characteristics

Forty-five patients with diabetes and with gastroparesis were enrolled; 42 completed 24 weeks of treatment. Patients were predominantly women, white, and overweight with mean diabetes durations of 21 years (Table 1). All patients with T1DM and T2DM were on insulin prior to enrollment. Nineteen of 32 patients (59%) with T1DM were on an insulin pump while 13 (41%) received other insulin regimens; 3 of 13 patients (23%) with T2DM were on insulin pump therapy and 10 (77%) were on other insulin programs. Details about insulin and other antidiabetic therapies used at screening by T1DM and T2DM patients are shown in Table A (Table A in S1 Tables). Glycemic control was poor with baseline hemoglobin A1c levels of 9.4%. Gastric emptying delays were moderately severe with 4 hour retention averaging 32%. Total GCSI, GCSI subscores, and PAGI-QOL scores reflected moderate-severe symptoms and quality of life impairments (Table 1). Additional details relating to individual symptom severity and PAGI-QOL domain scores are shown in Table B (Table B in S1 Tables).

**Table 1:** Patient characteristics at screening.

Category	Variable	All Patients (N = 45)	T1DM Patients (N = 32)	T2DM Patients (N = 13)	P Value (T1DM vs. T2DM)*
Demographic/clinical					
	Female	31 (69%)	21 (66%)	10 (77%)	0.72
	Age (yr)	45 (12)	42 (12)	53 (9)	0.001
	White	37 (82%)	27 (84%)	10 (77%)	0.67
	Hispanic	11 (24%)	6 (19%)	5 (38%)	0.25
	Known diabetes duration (years)	21 (11)	22 (12)	17 (10)	0.19
	Body mass index (kg/m^2^)	29 (8)	27 (6)	34 (10)	0.02
Medication use					
	Insulin (any regimen)	45 (100%)	32 (100%)	13 (100%)	1.00
	Continuous insulin pump therapy	22 (49%)	19 (59%)	3 (23%)	0.05
	Antidiabetic medications (other than insulin)	6 (13%)	2 (6%)	4 (31%)	0.05
	Proton pump inhibitors	32 (71%)	23 (72%)	9 (69%)	1.00
	Prokinetics	21 (47%)	15 (47%)	6 (46%)	1.00
	Antiemetics	24 (53%)	17 (53%)	7 (54%)	1.00
	Tricyclic antidepressants	9 (20%)	6 (19%)	3 (23%)	0.70
Metabolic					
	Hemoglobin A1c (%)	9.4 (1.4)	9.4 (1.3)	9.3 (1.6)	0.82
Gastric emptying					
	2 hour gastric retention (%)	63 (20)	62 (20)	66 (20)	0.57
	4 hour gastric retention (%)	32 (20)	31 (18)	36 (24)	0.52
Gastroparesis symptoms					
	Total GCSI score	29.3 (7.1)	28.8 (7.0)	30.7 (7.6)	0.44
	Total nausea/vomiting subscore	8.1 (4.2)	7.9 (4.1)	8.6 (4.4)	0.62
	Total fullness/early satiety subscore	14.1 (3.6)	14.0 (3.8)	14.4 (3.3)	0.76
	Total bloating/distention subscore	7.1 (2.3)	6.8 (2.3)	7.7 (2.3)	0.27
Quality of life					
	Mean PAGI-QOL score	2.4 (1.1)	2.6 (1.1)	1.8 (1.1)	0.06
Satiety testing					
	Water load (mL)	430 (207)	476 (208)	326 (168)	0.02
	Liquid nutrient (mL)	420 (258)	470 (263)	294 (202)	0.03

All values are either N (%) or mean (SD). Total GCSI score and subscale scores for nausea/vomiting, fullness/early satiety, and bloating/distention are sums of 9, 3, 4, and 2 components, respectively. PAGI-QOL domains are coded from 0 = lowest quality of life to 5 = highest quality of life. Mean PAGI-QOL score is the mean of the 5 domains.

* Based on Fisher’s exact test for categorical variables and unequal variance t-test for means.

### Primary outcome

#### Mild, moderate, and severe hypoglycemia episodes

Combined weekly rates of mild, moderate, plus severe hypoglycemic episodes confirmed by finger stick glucose testing were similar during the screening/run-in (1.9/week) and treatment phases (2.2/week) with a relative risk (RR) of 1.18 and 95% confidence interval (CI) of 0.85–1.64 (P = 0.33) (Table 2).

**Table 2:** Primary safety outcome during screening/run-in vs. treatment phases—Weekly combined mild, moderate, and severe hypoglycemic episodes.

Primary Outcome—Mild, Moderate, or Severe Hypoglycemic Episodes*	Screening/Run-in Phase* (N = 44)	Treatment Phase (N = 37)	Relative Risk (95% CI) Treatment vs. Screening/Run-In	P Value†
Number of events	594	1,604		
Number of patients with event	43	36		
Total person-weeks	320.6	735.3		
Rate per person-week	1.9	2.2	1.18 (0.85, 1.64)	0.33

* Includes Run-In phase data.

† Poisson regression using generalized estimating equations to account for within patient correlation across study phases.

### Secondary outcomes

#### Serious adverse events

Seven severe hypoglycemic events were adjudicated in 6 patients; 1 episode occurred during screening/run-in (0.1 event/patient-year) and 6 during treatment (0.3 event/patient-year) with an RR of 3.12 (95% CI 0.5–20.0) (P = 0.23) (Table 3).

**Table 3:** Serious adverse events.

Adverse Event		Screening/Run-in Phase (N = 45)	Treatment Phase (N = 42)	Relative Risk (95% CI) Treatment vs. Screening/Run-In	P Value†
Severe hypoglycemic events					
	Number of events	1	6		
	Number of patients with event	1	6		
	Rate per person-year	0.10	0.30	3.12 (0.5, 20.0)	0.23
Gastroparesis exacerbations‡					
	Number of events	9	11		
	Number of patients with event	4	7		
	Rate per person-year	0.88	0.55	0.50 (0.13, 1.96)	0.32
Other¶					
	Number of events	4	6		
	Number of patients with event	4	5		
	Rate per person-year	0.39	0.30	0.77 (0.19, 3.03)	0.69
Total					
	Number of events	14	23		
	Number of patients with event	8	16		
	Rate per person-year	1.37	1.14	0.77 (0.36, 1.64)	0.48

† Poisson regression using generalized estimating equations to account for within patient correlation across study phases.

‡ Includes nausea, vomiting, abdominal pain, diarrhea.

¶ Cholecystectomy, bilateral otitis media (2), hyperglycemia (3), rash, dizziness (2), retinal detachment.

Gastroparesis exacerbations occurred similarly during screening/run-in (9 events in 4 patients, 0.88/patient-year) and treatment (11 events in 7 patients, 0.55/patient-year) with an RR of 0.50 (95% CI 0.13–1.96) (P = 0.32) (Table 3). Other events unrelated to gastroparesis occurred similarly during screening/run-in and treatment (P = 0.69). This included an emergency department evaluation for severe hyperglycemia during the treatment phase in one patient with T1DM and one 5 day admission for severe hyperglycemia during the treatment phase in another patient with T1DM.

Clinical factors were compared in patients who did not versus did experience SAEs during treatment (Table C) (Table C in S1 Tables). Patients with SAEs reported higher overall GCSI scores, nausea/vomiting and early satiety/postprandial fullness subscale scores, and individual nausea, retching, and not able to finish meal scores than individuals who did not have SAEs (P≤0.04).

#### Glycemic control

**Fig 3:**
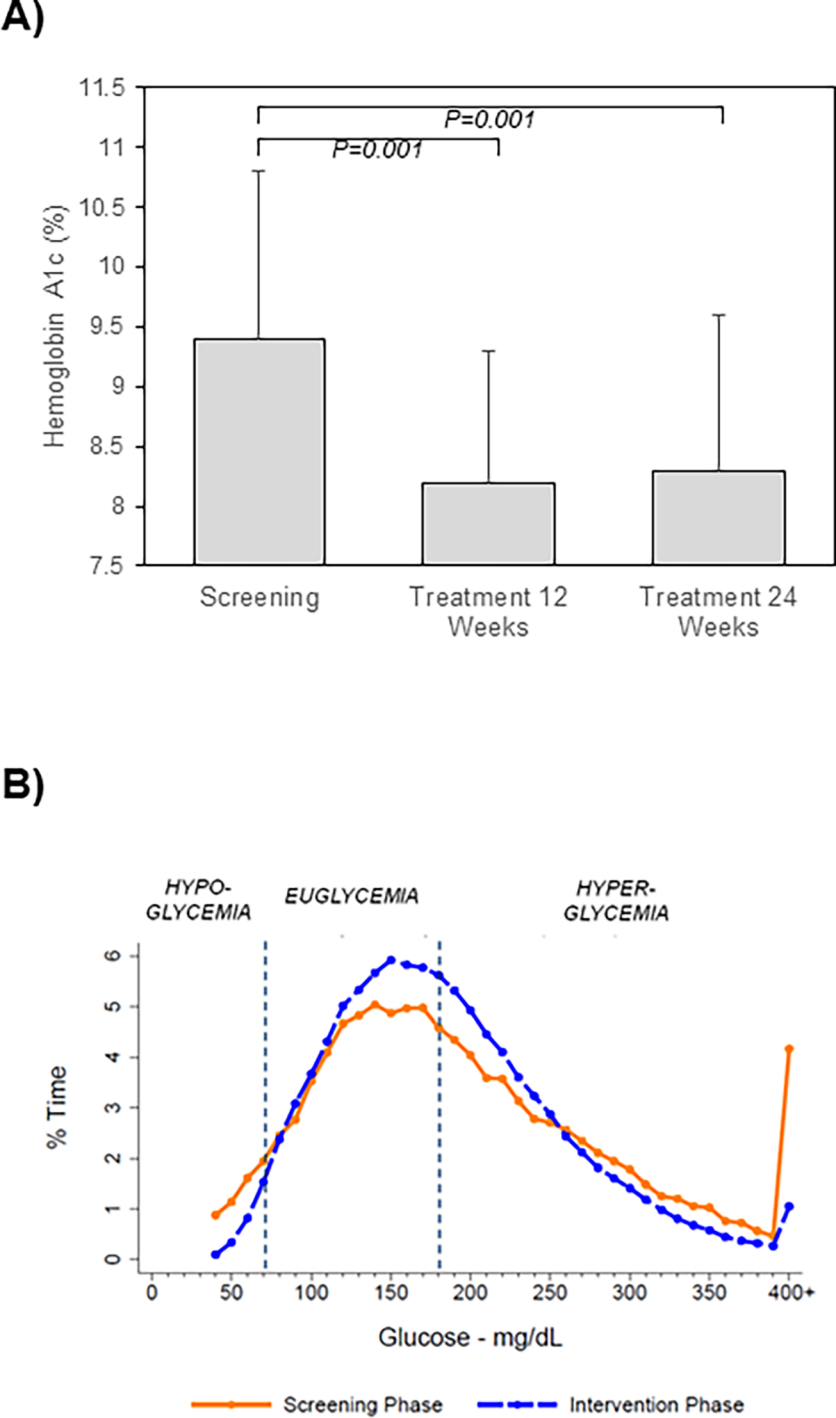
Effects of CSII plus CGM on glycemic parameters are shown. Treatment elicited significant reductions in hemoglobin A1c at 12 and 24 weeks of treatment compared to screening values (A). CGM readings revealed reductions in time in both hypo- and hyperglycemia with treatment at 24 weeks (blue) compared to the screening phase (orange) (B).

Hemoglobin A1c values decreased from 9.4±1.4% on screening to 8.2±1.1% at treatment week 12 (P = 0.001) and to 8.3±1.3% at week 24 (P = 0.001) (Fig 3A).

During screening/run-in, patients recorded a mean of 2,967±1,367 CGM readings over 14±6 days. This represented 74% of all possible readings during this phase. During treatment, a mean of 28,105±13,221 CGM readings per patient were acquired over 124±52 days. This was 70% of the total number of CGM readings possible during this phase. Mean CGM glucose levels decreased from 199±88 mg/dL to 184±71 mg/dL from screening to treatment (P = 0.005). Time in hypoglycemia (<50 mg/dL and <70 mg/dL) and hyperglycemia (>180 mg/dL and >300 mg/dL) were lower (P<0.05) and time in euglycemia was greater on treatment versus screening (P<0.01) (Table 4). Fig 3B displays GCM glycemia during screening and treatment illustrating reductions in glucose extremes with enhanced euglycemia. Percent of days free of excursions <50 mg/dL and >300 mg/dL were higher on treatment versus screening (P = 0.0002). Percent of days with excursions <50 mg/dL, <70 mg/dL, and >300 mg/dL but not >180 mg/dL were lower during treatment (P≤0.005) (Table 5).

**Table 4:** Frequencies of CGM glycemic excursions.

Glycemia Range	CGM Glucose Levels‡	Number of Glucose Excursions/Total Number of 5 Minute CGM Readings			Number of days with ≥1 Glucose Excursion/Total Number of Days with CGM Readings		
		Screening Phase	Treatment Phase*	P Value†	Screening Phase	Treatment Phase*	P Value†
Hypo- Glycemia							
	<50 mg/dL	1.2%	0.2%	<0.0001	16.2%	4.2%	<0.0001
	<70 mg/dL	3.9%	1.8%	<0.0001	34.8%	23.9%	0.002
Euglycemia							
	70–180 mg/dL	44.0%	52.0%	0.005	—	—	—
Hyper- glycemia							
	>180 mg/dL	52.1%	46.2%	0.04	96.4%	95.4%	0.23
	>300 mg/dL	14.2%	7.0%	<0.0001	59.2%	48.4%	0.02

* Excludes Run-In phase data.

† Logistic regression with generalized estimating equations (GEE) with independent working correlation to account for correlated data comparing screening phase to treatment phase.

‡ Includes glucose measurements from Interim Event form (N = 2 mild, N = 6 moderate, and N = 3 severe hypoglycemic events).

**Table 5:** Characteristics of glycemic excursions.

Glycemia Range	CGM Glucose Levels‡	Characteristic	Screening Phase	Treatment Phase*	P Value†
Hypoglycemia					
	<50 mg/dL				
		Total number	142	268	
		Length of excursions (minutes)—Median (IQR)	25 (15, 60)	28 (10, 55)	0.26
	<70 mg/dL				
		Total number	359	1761	
		Length of excursions (minutes)—Median (IQR)	30 (15, 80)	30 (15, 65)	0.17
Hyperglycemia					
	>180 mg/dL				
		Total number	1,323	11,784	
		Length of excursions (minutes)—Median (IQR)	80 (20, 270)	105 (30, 270)	0.32
	>300 mg/dL				
		Total number	760	4,028	
		Length of excursions (minutes)—Median (IQR)	45 (15, 135)	55 (20, 120)	0.18

* Excludes Run-In phase data.

† Logistic regression with generalized estimating equations (GEE) with independent working correlation to account for correlated data comparing screening phase to treatment phase.

‡ Includes glucose measurements from Interim Event form (N = 2 mild, N = 6 moderate, and N = 3 severe hypoglycemic events).

#### Gastroparesis symptoms

Total GCSI scores were lowered from 29.3±7.1 on screening to 21.6±9.6 at 12 weeks (P<0.0001) and to 21.9±10.2 at 24 weeks of treatment (P<0.0001) (Table 6). Nausea/vomiting, early satiety/postprandial fullness, and bloating/distention subscale scores improved at 12 and 24 weeks (P≤0.001). Improvements in individual symptoms are shown in Table D (Table D in S1 Tables).

**Table 6:** Effect of CSII plus CGM treatment on gastroparesis symptoms, quality of life, and meal tolerance.

Category	Variable	Screening Score Mean (SD)	12 Weeks Treatment		24 Weeks Treatment	
			Treatment Score Mean (SD)	P Value vs. Screening*	Treatment Score Mean (SD)	P Value vs. Screening*
Symptoms						
	Total GCSI score	29.3 (7.1)	21.6 (9.6)	<0.0001	21.9 (7.1)	<0.0001
	Total nausea/ vomiting subscore	8.1 (4.2)	4.9 (4.3)	<0.0001	5.0 (4.3)	<0.0001
	Total fullness/ early satiety subscore	14.1 (3.6)	10.9 (4.3)	<0.0001	11.3 (4.7)	0.0008
	Total bloating/ distention subscore	7.1 (2.3)	5.8 (2.9)	0.0009	5.5 (3.0)	0.0002
Quality of life						
	Mean PAGI-QOL score	2.4 (1.1)	3.0 (1.1)	<0.0001	3.1 (1.1)	<0.0001
Satiety testing						
	Water load (mL)	430 (207)	432 (233)	1.00	413 (238)	0.46
	Liquid nutrient (mL)	420 (258)	417 (226)	0.47	487 (312)	0.05

All values are either N (%) or mean (SD).

* Based on paired t-test of mean change = 0.

#### Quality-of-life

Total PAGI-QOL scores improved from 2.4±1.1 on screening to 3.0±1.1 after 12 weeks of treatment (P<0.0001) and to 3.1±1.1 after 24 weeks of treatment (P<0.0001) (Table 6). All individual PAGI-QOL domain scores improved at 12 and 24 weeks (P≤0.02) (Table D) (Table D in S1 Tables).

#### Meal tolerance

Compared to screening, tolerance of water ingestion was unchanged by treatment (P = 1.00 at 12 weeks; P = 0.46 at 24 weeks) (Table 6). Liquid nutrient tolerance increased from 420±258 mL on screening to 487±312 mL at 24 weeks of treatment (P = 0.05).

### Diabetes subtype comparisons

Post-hoc analyses contrasted outcomes in T1DM versus T2DM. Mild, moderate, and severe hypoglycemic episodes were lower during screening versus treatment in T2DM with an RR of 2.0 (95% CI 1.1, 3.3)(P = 0.03), but were unchanged by treatment in T1DM (P = 0.95) (Table E) (Table E in S1 Tables). Hemoglobin A1c decreased from 9.3±1.6 at screening to 7.4±1.2 at 24 weeks in T2DM and from 9.4±1.3 at screening to 8.8±1.2 at 24 weeks in T1DM. These improvements were greater in T2DM (-2.0±2.1%) versus T1DM (-0.7±1.1%)(P = 0.002) (Table F) (Table F in S1 Tables). Total GCSI scores decreased from 30.7±7.6 at screening to 28.0±8.9 at 24 weeks in T2DM and from 28.8±7.0 at screening to 19.1±9.6 at 24 weeks in T1DM. These improvements at 24 weeks were greater in T1DM (-9.3±9.4) than T2DM (-2.0±7.0)(P = 0.01). Early satiety/fullness and bloating/distention subscale scores and individual nausea, stomach fullness, feeling excessively full, bloating, and stomach visibly larger scores decreased more in T1DM versus T2DM (P≤0.03). Other SAEs and changes in PAGI-QOL scores and water and nutrient tolerance were similar between subtypes.

## Discussion

CSII plus CGM (i) did not significantly increase hypoglycemia episodes (ii) improved hemoglobin A1c and CGM glycemia profiles, (iii) improved symptoms and quality-of-life, and (iv) enhanced liquid nutrient tolerance in patients with poorly controlled diabetes and gastroparesis.

Because of concerns that intensifying insulin therapy in diabetic gastroparesis might cause hypoglycemia due to mismatches consequent to delayed nutrient absorption, our primary outcome was to compare combined weekly mild, moderate, and severe hypoglycemic episodes before and during CSII plus CGM [27]. Classical models of insulin administration to patients without gastroparesis are designed to match postprandial nutrient absorption to short-acting insulin analog pharmacokinetics, which may be inappropriate in diabetic gastroparesis. Dual wave CSII features permit delivery of second insulin waves and/or temporary basal rate increases to address this mismatch [28, 29]. Treatment did not increase combined mild, moderate, and severe hypoglycemia rates. However, GLUMIT-DG was shorter in duration than DCCT or the Action to Control Cardiovascular Risk in Diabetes (ACCORD) trials [30, 15]. Nevertheless, the implication of our findings is that CSII plus CGM can be implemented in diabetic gastroparesis without worsening overall hypoglycemia risks.

A secondary feasibility outcome was to verify that intensive regimens improve glycemic control in diabetic gastroparesis. Our protocol improved hemoglobin A1c and CGM profiles, including reduced hypo- and hyperglycemic excursions which were pronounced for very low (<50 mg/dL) and high (>300 mg/dL) glucose levels. The 1.1% hemoglobin A1c decreases persisted for 24 weeks, but were less than in DCCT (1.9%), ACCORD (1.9%) and the previous diabetic gastroparesis study (1.8%) [15, 17, 30]. It should be emphasized that GLUMIT-DG was not designed to achieve specific hemoglobin A1c or CGM glycemia profiles.

Secondary efficacy outcomes of GLUMIT-DG defined if intensive regimens are potentially effective in improving symptoms, quality of life, and meal tolerance. CSII plus CGM reduced overall GCSI and PAGI-QOL scores over 24 weeks. Our findings expand on a study in which CSII reduced gastroparesis-related hospitalizations in T1DM patients [17]. That investigation was substantially different from GLUMIT-DG as it was retrospective and did not quantify emptying, symptoms, or quality-of-life. In the only other study to examine symptom responses to glycemic control, postprandial fullness was identical in year 13/14 of the Epidemiology of Diabetes Interventions and Complications study in patients initially randomized to intensive versus conventional insulin therapies in DCCT [9]. Liquid nutrient tolerance was enhanced by CSII plus CGM over 24 weeks, suggesting treatment may improve gastric functions like fundic accommodation [23]. Others have shown that improving glycemia normalizes delayed emptying in women with newly diagnosed T2DM without gastroparesis [31]. However, our results contrast with an investigation in T2DM gastroparesis reporting no emptying acceleration with intensive glycemic regimens that decreased hemoglobin A1c from 10.6% to 9.3% [18]. Longer term studies over 12–25 years observed no emptying stimulation despite improved hemoglobin A1c in gastroparesis patients [6, 7, 32]. Acute hyperglycemia disrupts gastric motor, myoelectrical, and sensory function in patients with diabetes and healthy controls [33, 34, 35, 36]. Whether the enhanced meal tolerance elicited by treatment in GLUMIT-DG is a consequence of reversing chronic diabetes related-gastric neuromuscular impairment or resolution of inhibitory effects of acute hyperglycemia is uncertain.

Risk factors for SAEs were identified by analyzing patient characteristics and symptoms at screening. Diabetes subtype and glycemic control did not influence SAE occurrence, but baseline nausea and early satiety intensity correlated with SAE frequencies. Although patient numbers were small and SAEs were infrequent, this suggests patients with diabetes with worse nausea or early satiety may need more aggressive surveillance while adopting intensive insulin therapies.

In post-hoc analyses, GLUMIT-DG results were not uniform between diabetic subtypes. Hypoglycemic events increased and hemoglobin A1c decreased more in T2DM, while symptom improvements on treatment were greater with T1DM. However, GLUMIT-DG was not powered to assess differences between diabetes subtype and results may have been influenced by small sample sizes. In particular, the T2DM cohort was smaller and exhibited high rates of insulin use, low rates of oral anti-diabetic medication use, and exaggerated impairments in nutrient tolerance, raising concerns about whether our T2DM findings can be generalized to T2DM patients managed only with oral agents.

Our study had limitations. It was powered on the primary outcome of combined weekly episodes of mild, moderate, and severe hypoglycemia. Although GLUMIT-DG had the largest recruitment to date of well-characterized patients with diabetes and with gastroparesis into such a rigorous, standardized protocol, our sample size and treatment durations were somewhat modest to compare treatment effects on severe hypoglycemic episodes and efficacy outcomes. Although severe hypoglycemic events were numerically greater on CSII plus CGM, these differences did not approach statistical significance. Severe hypoglycemia rates on treatment in GLUMIT-DG (0.3/patient-year) were lower than the DCCT intensive treatment cohort consistent with safe use of CSII plus CGM in these patients [16]. Furthermore, patients had diverse backgrounds prior to study enrollment with respect to pre-enrollment insulin regimens with a subset already on an insulin pump and the remainder receiving either combined long-acting insulin and/or sliding scale short acting agents. However, sample sizes were too small to determine if this pre-enrollment heterogeneity influenced CSII plus CGM treatment effects on primary and secondary study outcomes. Glycemia was not assessed on baseline gastric emptying assessment, and treatment effects on symptoms over 24 weeks were not related to emptying acceleration. However as symptoms correlate poorly with emptying rates and gastric responses to prokinetics, this information may be less important [4, 37]. GLUMIT-DG did not use a control group which may have impacted interpretation of efficacy outcomes. This study was not a randomized controlled trial (RCT), but it should be recognized that this work is a necessary first step before an RCT can be considered in this disorder. Consequently, symptom reductions with CSII plus CGM may reflect placebo responses rather than a consequence of better metabolic control. Consideration should be given to future trials randomizing patients to the existing GLUMIT-DG protocol versus more aggressive regimens to normalize hemoglobin A1c.

## Conclusions

In conclusion, CSII and CGM appeared to be safe over 24 weeks with no significant increases in overall hypoglycemic events in patients with poorly controlled diabetes and gastroparesis. Treatment also improved glycemic profiles on CGM, reduced hemoglobin A1c levels, and improved gastroparesis symptoms, quality-of-life, and meal tolerance. These pilot observations provide evidence for including more aggressive insulin regimens in the management of diabetic gastroparesis.

## Supporting information

S1 File This file includes the TREND checklist. (PDF)

S2 File This file includes Institutional Review Board approval information for each site. (PDF)

S3 File This file includes the initial study protocol dated August 12, 2009. (PDF)

S4 File This file includes the amended study protocol dated March 16, 2011. (PDF)

S5 File This file includes the amended study protocol dated April 16, 2013. (PDF)

S1 Tables This file includes Tables A, B, C, D, E, and F.
(PDF)
