# Supplementary material for: Glucose sensor-augmented continuous subcutaneous insulin infusion in patients with diabetic gastroparesis: An open-label pilot prospective study
Source: PLoS One. 2018 Apr 13;13(4):e0194759. doi: 10.1371/journal.pone.0194759 (PMC5898706; doi:10.1371/journal.pone.0194759)
Supplement: S2 File — (PDF) [file pone.0194759.s002.pdf]

Institutional Review Board (IRB) Approvals at All Sites

The initial study protocol was approved by the ethics committees and Institutional Review Board (IRB) committees at each site (except MetroHealth) within the GpCRC in 2009-10 before the study began at that site. A second amended study protocol was approved by the ethics committees and IRB committees at all sites (except MetroHealth) in 2011. MetroHealth Regional joined the study in 2013; a third amended study protocol was approved by the ethics committees and IRB committees at all sites in 2013. Names of institutional review boards (and initial approval dates) are as follows: MetroHealth Institutional Review Board (July 22, 2013), Wake Forest University Health Sciences Institutional Review Board (October 12, 2009), University of Michigan Medical School Institutional Review Board (January 21, 2010), Johns Hopkins Bloomberg School of Public Health Institutional Review Board Office (October 30, 2009), Temple University Research Review Committee (February 16, 2010), Stanford University Panel on Medical Human Subjects (June 11, 2010), California Pacific Medical Center Institutional Review Board (April 1, 2010), Texas Tech Health Sciences Center Institutional Review Board for the Protection of Human Subjects (May 26, 2010), and University of Mississippi Medical Center Institutional Review Board (August 3, 2010).
