# Supplementary material for: Glucose sensor-augmented continuous subcutaneous insulin infusion in patients with diabetic gastroparesis: An open-label pilot prospective study
Source: PLoS One. 2018 Apr 13;13(4):e0194759. doi: 10.1371/journal.pone.0194759 (PMC5898706; doi:10.1371/journal.pone.0194759)
Supplement: S3 File — (PDF) [file pone.0194759.s003.pdf]

GpCRC

Gastroparesis
Clinical Research Consortium

Pilot Study of the Safety, Feasibility, and Potential Efficacy of Continuous Glucose Monitoring and Insulin Pump Therapy in Diabetic Gastroparesis (GLUMIT-DG)

Protocol

12 Aug 2009

Contents

Design synopsis ... 3

1. Objectives ... 7

2. Background and significance ... 8
   - 2.1. Introduction ... 8
   - 2.2. Diabetic gastroparesis ... 8
   - 2.3. Continuous glucose monitoring system (CGMS) ... 9
   - 2.4. Insulin pump ... 9

3. Study design ... 10
   - 3.1. Design overview ... 10
   - 3.2. CGMS training through the screening period ... 11
   - 3.3. Insulin pump training ... 12
   - 3.4. Standard treatment recommendations ... 13

4. Patient selection ... 14
   - 4.1. Recruitment ... 14
   - 4.2. Inclusion criteria ... 14
   - 4.3. Exclusion criteria ... 15
   - 4.4. Run-in phase ... 15

5. Protocol procedures ... 16
   - 5.1. Visit schedule overview ... 16
   - 5.2. Screening and baseline data collection overview ... 16
   - 5.3. Run-in visits with insulin pump training ... 20
   - 5.4. Enrollment ... 21
   - 5.5. Follow-up visits ... 21
   - 5.6. Standardized questionnaires ... 28
   - 5.7. Specimen repository ... 29

6. Safety monitoring ... 30
   - 6.1. Safety issues ... 30
   - 6.2. Adherence and retention ... 34
   - 6.3. Management of diabetes ... 34
   - 6.4. Adverse event reporting ... 34
   - 6.5. Reporting and adjudication of serious adverse events ... 36

7. Statistical design and analysis ... 38
   - 7.1. Hypotheses ... 38
   - 7.2. Outcome measures ... 38
   - 7.3. Statistical issues ... 40
   - 7.4. Interim analysis ... 41

8. Human subjects issues ... 43

9. References ... 44

10. Appendices ... 46
   - 10.1. Participating centers ... 47

GLUMIT-DG Protocol

10.2. Data collection schedule 48
10.3. Whole blood draw schedule 49
10.4. Glossary 50

Design synopsis

Title
- Pilot Study of the Safety, Feasibility, and Potential Efficacy of Continuous **Glu**cose **M**onitoring and **I**nsulin Pump **T**herapy in **D**iabetic **G**astroparesis (GLUMIT-DG)

Sponsor
- NIDDK

Type of study
- Multicenter, uncontrolled, open label treatment
- Safety and feasibility

Objective
- To assess the safety, feasibility, and potential (uncontrolled) efficacy of continuous glucose monitoring (CGMS) in conjunction with an insulin pump to improve glycemic control for treatment of type 1 and type 2 diabetic patients with gastroparesis

Population
- Patients aged 18 - 70 years old at registration with mild to moderate symptoms of gastroparesis and a diagnosis of type 1 or type 2 diabetes mellitus for at least 2 years.

Study duration – per patient
- A minimum of 4 weeks and up to 16 weeks of screening and baseline assessments prior to enrollment
- 24 weeks of open label treatment and follow-up after enrollment

Study duration – per calendar time
- Recruitment phase: September 2009 - June 2010
- Follow-up phase: September 2009 - December 2010

Sample size and statistical analysis of primary outcome
- A total of 40 patients will be enrolled in this pilot study
- Rationale for sample size
  - Primary safety outcome: weekly frequency of mild, moderate, or severe hypoglycemic episodes by CGMS
  - Assumed mean weekly mild, moderate, or severe hypoglycemic episodes by CGMS: 0.6
  - Assumed SD for weekly mild, moderate, or severe hypoglycemic episodes by CGMS: 0.8

- Assumed intra-class correlation between baseline and on-treatment weekly mild, moderate, or severe hypoglycemic episodes by CGMS: 0.6
- Estimated variance of change from baseline (a minimum of 4 weeks up to 16 weeks) to on-treatment (24 weeks) mean weekly mild, moderate, or severe hypoglycemic episodes (estimated from SD and intraclass correlation, accounting for 3:1 ratio of on-treatment to baseline period lengths): 0.41
- Type I error: 0.05 (1-sided for equivalence)
- Power: 0.9
- Expected percent increase from baseline in mean weekly mild, moderate, or severe hypoglycemic episodes during the treatment period with CGMS while using the glucose pump: 0%
- Equivalence limit (maximum percent increase in mean weekly mild, moderate, or severe hypoglycemic episodes): 35% (0.6 vs. 0.81 episodes per week)
- Method for sample size calculation: One sample test for equivalence (Chow, Shao and Wang, Sample Size Calculations In Clinical Research, Taylor & Francis, NY, 2003: p. 52-53)
- Software: Centre for Clinical Trials, Chinese University of Hong Kong -- http://www.cct.cuhk.edu.hk/stat/mean/osm_equivalence.htm
- Calculated sample size: N = 37

- Assess sample size assumptions after 10 patients have completed the study and adjust the total sample size target of 40, if needed

Number of clinical centers

- 7 (two lead clinics and 5 additional clinical sites added later)

Inclusion criteria

- Age 18 - 70 years old at registration
- Type 1 or Type 2 diabetes mellitus for at least 2 years
- Symptoms of gastroparesis (nausea, vomiting, early satiety, bloating, fullness, discomfort) for at least 1 year prior to registration with a Gastroparesis Cardinal Symptom Index (GCSI) score of ≥18
- Delayed gastric emptying on gastric scintigraphy within 1 year of registration, defined as greater than 60% retention at 2 hours or greater than 10% retention at 4 hours
- Hemoglobin A1c of at least 8.0% at registration regardless of current therapy. Individuals already receiving diabetes therapy via an insulin pump will be eligible for study participation if, in the opinion of the investigators, he/she may acquire additional benefit from continuous glucose monitoring that might improve glycemic control
- Normal upper endoscopy within 1 year of registration
- No clinical or imaging evidence of obstruction
- Successful mastering of use of CGMS during the run-in period

Exclusion criteria
- Prior gastric surgery including fundoplication
- Other systemic disease potentially causative of gastrointestinal symptoms
- Acute or chronic renal insufficiency with creatinine >1.5 mg/dL
- Psychiatric disease or eating disorder
- Contradiction to gastric emptying breath test such as a known allergy to egg, wheat, or algae
- Pregnancy
- Any other condition which, in the opinion of the investigators, would impede compliance or hinder completion of the study

Outcome measures
- ***Primary:*** The primary outcome measure is defined as the frequency and extent of mild or moderate hypoglycemic episodes (defined as a glucose level less than 70 mg/dL obtained from the CGMS and finger stick data) and absolute number of severe hypoglycemic episodes at 12 and 24 week follow-up visits while using a combination of CGMS and insulin pump therapy.

- **Secondary outcome measures** will be defined to address the following:
  Effects of CGMS and insulin pump therapy on:
  - Hemoglobin A1c at baseline, 12, and 24 week follow-up visits
  - Percent time of day in hypoglycemia, euglycemia, or hyperglycemia
  - Glycemia variability (blood glucose rate of change)
  - Marked hyperglycemia index
  - GCSI, PAGI-SYM and PAGI-QOL scores at baseline, 12, and 24 week follow-up visits
  - Gastric emptying rate at baseline, 12, and 24 week follow-up visits
  - Maximal volume of water and Ensure consumed during satiety tests at baseline, 12, and 24 week follow-up visits
  - Percent tachygastria, bradygastria and normal 3 cpm activity during EGG and satiety testing at baseline, 12, and 24 week follow-up visits
  - Vagal cholinergic and sympathetic adrenergic functions at baseline, 12, and 24 week follow-up visits

Recruitment
- 20 type 1 and 20 type 2 diabetics with gastroparesis will be enrolled at 7 clinical centers
- 10 month period
- Enrollment rate of 0.57 patients per clinical center per month

Duration of open label treatment and follow-up
- 24 weeks

Visit schedule

- Screening into the study: enrollment should occur within 16 weeks of registration
  - During the CGMS training, prospective participants will learn how to wear a sensor and how to collect CGMS data
  - After successful completion of the CGMS training during the run-in period, prospective participants will learn how to use CGMS to guide insulin pump therapy
- Run-in visits:
  - At least 4 visits to receive training on use of an insulin pump in conjunction with CGMS
- Treatment and follow-up phase
  - Every 4 weeks after enrollment throughout the 24 week open label treatment study

Safety monitoring

- The NIDDK-appointed DSMB will perform interim monitoring of the accumulating GLUMIT-DG study data for patient safety and potential (uncontrolled) efficacy according to the Data and Safety Monitoring Plan (DSMP) for the GLUMIT-DG study

1. Objectives

The principal objective of this multicenter, uncontrolled, open label treatment study is to assess the safety of CGMS in guiding insulin pump therapy for 24 weeks by measuring mild, moderate, and severe hypoglycemic episodes in patients with type 1 and type 2 diabetes and gastroparesis.

Other objectives include:

- To determine the feasibility of achieving improved glycemic control with CGMS guided insulin pump therapy in patients with diabetes and gastroparesis.
- To determine the efficacy of CGMS guided insulin pump therapy for 24 weeks on symptoms of gastroparesis, gastroparesis-related quality of life, gastric emptying, satiety, gastric myoelectrial activity, and autonomic function in diabetics with gastroparesis.

---

2. Background and significance

2.1. Introduction

Gastroparesis is a disorder of gastric neuromuscular sensory and motor function that affects patients in the prime of their life. Gastroparesis is a devastating disease affecting predominantly young women (females outnumber males by a ratio of 4:1, with an average 34 years of age)[1]. The symptomatic profile of gastroparesis includes nausea (90% of patients), vomiting (>80%), pain (~50%), early satiety (60%), and bloating (75%) and can vary in both the combination of symptoms and their severity[1]. Because of its chronic, and often intractable nature, the disorder has a tremendous impact on both patients and society at large. Gastroparesis remains difficult to treat, in large part because of the lack of knowledge of the underlying pathophysiology of this disease. Several factors, in particular, have impeded the progress in this field including the paucity of patients seen by any one center, the absence of uniform diagnostic criteria, the lack of generally available, reliable methods for physiological testing and the inaccessibility of tissue for histopathological correlation.

The Gastroparesis Clinical Research Consortium (GpCRC) is a cooperative network of six clinical centers and one Data Coordinating Center (DCC) supported by the National Institute of Diabetes and Digestive and Kidney Diseases (NIDDK), through the mechanism of RFA-DK-05-004, established in 2006. Clinical centers are responsible for proposing protocols, participating in their overall development, recruiting patients, conducting the research, and disseminating research findings. The individual clinical centers participate in a cooperative and interactive manner with one another and with the DCC in all aspects of the GpCRC. The DCC supports protocol development; provides sample size calculations, statistical expertise, forms, and data analysis; supports manuscript preparation; and provides overall study coordination and quality assurance, including coordination of the activities of the Data and Safety Monitoring Board, the Steering Committee and other standing committees.

2.2. Diabetic gastroparesis

It is well-established in the literature that optimal control of blood sugar in patients with long-standing diabetes mellitus slows the progression of several of the systemic complications of the disease, including peripheral neuropathy and renal disease[2]. Gastrointestinal manifestations are prevalent in individuals with diabetes, especially in those who have had poorly controlled disease for 10 or more years[3]. Nausea, vomiting, fullness, early satiety, bloating, discomfort, and altered glycemic control represent the symptoms of diabetic gastropathy[4,5]. Many individuals with diabetic gastropathy exhibit delays in gastric emptying of solid meals and are considered to have gastroparesis[6,7]. However, studies of the effects of hyperglycemia on gut function have been limited

to acute settings in which some parameter of muscular, myoelectrical, or sensory function is modulated by acute experimental changes in blood glucose.

In diabetics, induction of hyperglycemia slows both solid and liquid phase gastric emptying, blunts phasic antral contractions, and leads to generation of gastric slow wave dysrhythmias such as tachygastria [8,9]. In healthy non-diabetic volunteers, acute hyperglycemia also induces tachygastria and blunts antral contractility [10]. Acute hyperglycemia also increases gastric sensitivity to mechanical stimulation and reduces responses to prokinetic drugs [11]. On the basis of these acute studies, gastroenterologists routinely recommend optimal glycemic control to minimize gastrointestinal consequences of poorly controlled diabetes. However to date, there remains no hard data from well-controlled, long-term trials to validate this recommendation.

2.3. Continuous glucose monitoring system (CGMS)

Recently, commercially available continuous glucose monitors have become available to track interstitial glucose levels in real time in patients with diabetes. This method has significant potential to enhance glycemic control in diabetics with frequent hypoglycemic events. Indeed, continuous monitoring detects a high number of unsuspected hypoglycemic events which are unrecognized by the patient with the traditional finger stick method [12]. Furthermore, this method has been shown to reduce mean hemoglobin A1c values by 0.4% within a 12-week period[13]. From a safety standpoint, continuous glucose monitoring increases the detection rate of nocturnal hypoglycemia in type 1 diabetics [14].

This study will determine the safety, feasibility, and potential efficacy of using a continuous glucose monitoring system to guide insulin pump therapy in patients with type 1 and type 2 diabetes complicated with gastroparesis. Along with the measurement of interstitial glucose concentrations with CGMS, patients in this study will be instructed to perform measurements of blood glucose levels four times daily using a finger stick method with a One Touch® Ultra glucose meter. The finger stick glucose results will be used for calibration of the CGMS and also for glucose monitoring as an additional guide for insulin therapy. The results from both glucose measuring methods will be used to guide insulin infusion with a continuous subcutaneous insulin infusion system (insulin pump).

2.4. Insulin pump

Along with the use of a CGMS, patients will use the Minimed Paradigm® Insulin Pump (Medtronic) to achieve optimal blood glucose control. The rate of basal insulin infusion and the amount of insulin for meals as well as any extra amount of insulin needed for a glucose reading that exceeds 180 mg/dL will be adjusted using real time glucose measurements obtained from the CGMS. Participants will receive extensive training on the use of both CGMS and the insulin pump which will replace the current diabetes treatment. Optimization of insulin pump treatment will take place for those who are already using an insulin pump prior to GLUMIT-DG registration. The parameters of the pump will initially be programmed according to parameters based on the patient's type of diabetes, food intake, and body weight. Special precautions will be built into bolus insulin administration for meals since all patients will have gastroparesis.

3.1. Design overview

GLUMIT-DG is a multicenter, uncontrolled open label treatment study assessing the safety, feasibility, and potential efficacy of CGMS in conjunction with insulin pump therapy for patients with type 1 and type 2 diabetes and gastroparesis. Participants will include patients enrolled in the Gastroparesis Registry qualifying for GLUMIT-DG and other patients with diabetes and gastroparesis referred to study physicians, or patients from the investigators' clinics. Twenty patients with type 1 diabetes and twenty patients with type 2 diabetes will be enrolled into GLUMIT-DG study. Screening for eligibility, collection of baseline data, CGMS training, and the run-in period for baseline glycemic assessments will span up to 16 weeks after obtaining informed consent for participation in the study. After successful completion of the run-in period, patients will be enrolled in the study, and use CGMS coupled with insulin pump therapy to optimize blood glucose levels while determining the frequency of hypoglycemic events for 24 weeks. After study completion, patients will be given the option of returning to or participating in the Gastroparesis Registry. A schematic of the study design is presented below:

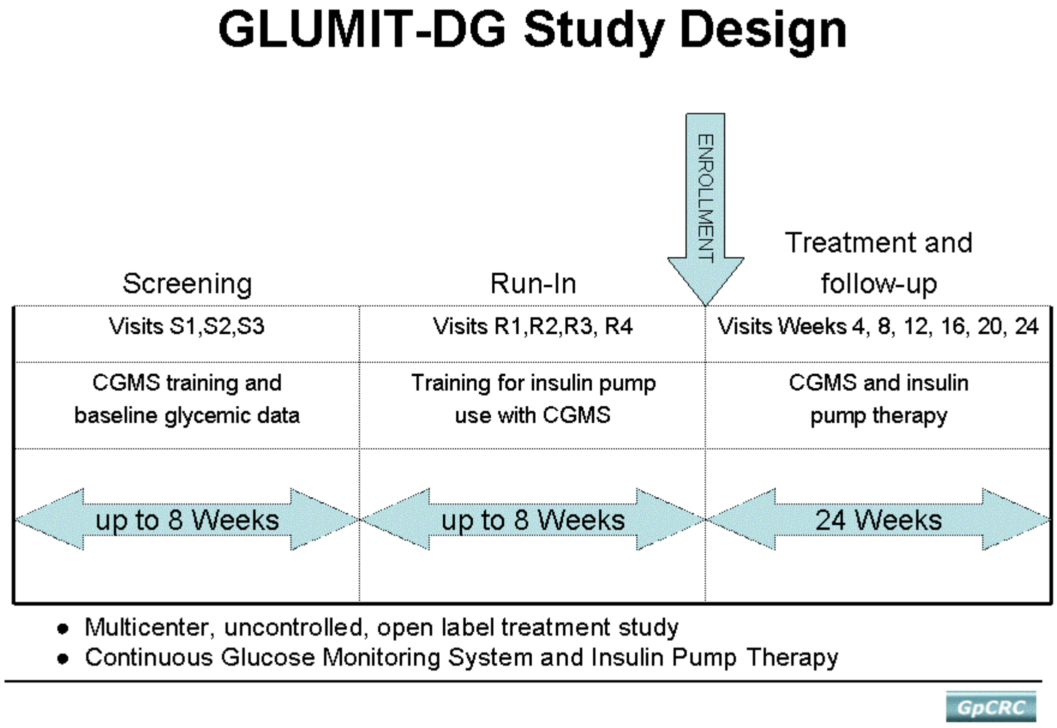

GLUMIT-DG Protocol

The primary outcome measure of GLUMIT-DG relates to the safety (hypoglycemic episodes) of the use of CGMS to guide insulin pump therapy in patients with diabetes and gastroparesis. Secondary analyses will include: the effects of the use of CGMS to guide insulin pump therapy on hemoglobin A1c levels, glycemic variability (blood glucose rate of change), marked hyperglycemia index, Patient Assessment of Upper Gastrointestinal Disorders- Symptoms (PAGI-SYM), Patient Assessment of Upper Gastrointestinal Disorders- Quality of Life (PAGI-QOL) scores, gastric emptying rate, maximal volume of water and caloric drink consumed during satiety tests, percent tachygastria during electrogastrogram (EGG), and vagal cholinergic and sympathetic adrenergic functions.

3.2. CGMS training through the screening period

Patients will initially be instructed by the diabetologist and the diabetes educator on use of the MiniLink™ REAL-Time Transmitter (Medtronic) CGMS. Patients will then be requested to obtain glycemic data using the CGMS during a period lasting at least 2 weeks (or potentially 4 weeks depending on a patient's performance level). Patients will also be requested to perform measurements of their blood glucose levels 4 times daily using a standard finger stick method with a One Touch® Ultra glucose meter. Blood glucose levels obtained from the finger stick method will be used to calibrate the CGMS and also for blood glucose monitoring during this CGMS learning phase. Data obtained from the CGMS during this training period will not be used for diabetes management. A successful CGMS training will be defined as the acquisition of continuous recording of glycemic data for at least 216 hours (9 days) or greater during a 2 week period. If patients cannot successfully master the CGMS and collect the required duration of data, they will be offered an additional 2 week period to demonstrate proficiency in the use of CGMS to collect the sufficient amount of data (216 hours or more). If the patient is again unsuccessful, he/she will be considered a screen failure. Along with showing CGMS usage capability, patients must perform 4 blood glucose tests (finger sticks) daily, as another indicator of proficiency and compliance in order to continue in the screening phase of the study. Additionally, patients with type 1 diabetes will also be trained on the use of serum ketone meters.

The following additional glycemic data will be calculated from the CGMS prior to enrollment in the GLUMIT-DG study:

- Percent time of the day during which prevailing glucose levels are within euglycemic boundaries (80-180 mg/dL).
- Low blood glucose index (LBGI) reflecting the frequency and extent of hypoglycemic episodes.
- High blood glucose index (HBGI) reflecting the frequency and extent of hyperglycemic episodes.
- Glycemia variability standard deviation of blood glucose rate of change.
- Number of episodes of hypoglycemia (glucose <70 mg/dL, mild or severe) per week recorded by CGMS and also but not captured by the standard finger stick approach.

GPCRC\GLUMIT-DG\Protocol\Manall_87
3:12 pm Wednesday, August 12, 2009/klc

Subsequent to the screening period, the CGMS will continue being used throughout the study to monitor blood glucose levels for each patient. Further training on usage of the CGMS will continue to be given during the run-in visits. Both the diabetes educator and the diabetologist will assist in patient troubleshooting of the CGMS beginning with the screening period and for the duration of the study.

3.3. Insulin pump training

Patients who have signed an informed consent statement, met the eligibility criteria, and have successfully demonstrated proficiency with using the CGMS during the training period (up to 8 weeks) will continue into the run-in period for further training of CGMS now adding the use of an insulin pump. Patients will receive training on how to use the CGMS data for the optimization of insulin administration using the pump. After successful completion of the run-in phase, patients will be enrolled into the GLUMIT-DG study for 24 weeks for control of glycemia using the CGMS to guide insulin pump treatment.

The initial insulin pump parameters will be set as follows:

For those patients who were not using an insulin pump prior to screening, parameters will be set initially based on type of diabetes and body weight (BW in kilograms):

For Type 2 diabetes:

1. Initial hourly basal insulin rate will be estimated as (BW x 0.4)/24 units of insulin
2. The initial carbohydrate to insulin ratio for meal boluses will be estimated as 450/(BW x 0.84) (grams of carbohydrate per unit insulin)
3. Initial correction factor for fasting glycemia >110 mg/dL, postprandial >140 mg/dL will be estimated as 1700/(BW x 0.8) for capillary glucose drop per unit insulin

For Type 1 diabetes:

1. Initial hourly basal rates will be estimated as (BW x 0.2)/24 units of insulin
2. Initial meal boluses using a carbohydrate to insulin ratio will be estimated as 450/(BW x 0.2) (grams of carbohydrate per unit insulin)
3. Initial correction factor for fasting glucose >110 mg/dL, postprandial >140 mg/dL will be estimated as 1400/(BW x 0.4) for capillary glucose drop per unit insulin

Alternatively, for those patients who are using a Basal-Bolus approach to control their Type 1 diabetes, the insulin pump may be programmed to use their current basal dose and carbohydrate to insulin ratios as well as correction factor.

For those patients who are already on insulin pumps, the insulin parameters will be adjusted based on the best prediction of needs using results from the baseline (run-in period) CGMS and finger stick data.

Overall the targets for glycemia management will be:

1. Fasting glucose: 80-120 mg/dL
2. Postprandial excursions: 100-180 mg/dL

GPCRC\GLUMIT-DG\Protocol\Manall_87
3:12 pm Wednesday, August 12, 2009/klc

3.4. Standard treatment recommendations

During the screening and run-in period that may span up to 16 weeks prior to enrollment into the GLUMIT-DG study, patients will receive a standardized set of recommendations to manage their gastroparesis symptoms including dietary modification, weight loss if needed, and exercise. In addition to dietary modification, patients may use antiemetic, prokinetic, or pain medications as appropriate for their gastroparesis symptom management. These recommendations have been prepared by the GpCRC Steering Committee as standard of care in the management of patients with diabetic gastroparesis (see Standard Operating Procedures IV: Standard of Care for Patients with Diabetic Gastroparesis). This will help ensure that patients receive standard of care treatment for gastroparesis.

GPCRC\GLUMIT-DG\Protocol\Manall_87
3:12 pm Wednesday, August 12, 2009/klc

4. Patient selection

4.1. Recruitment

Approximately 40 patients will be recruited from the seven clinical sites of the GpCRC (averaging 5.7 patients per center) over a 10-month period:

- Stanford University, Palo Alto, CA (PI: P. Jay Pasricha, MD)
  - California Pacific Medical Center, San Francisco, CA (Co-PI: William Snape, MD)
- Temple University, Philadelphia, PA (PI: Henry Parkman, MD)
- Texas Tech University, El Paso, TX (PI: Richard McCallum, MD)
- University of Michigan, Ann Arbor, MI (PI: William Hasler, MD)
- University of Mississippi, Jackson, MS (PI: Thomas Abell, MD)
- Wake Forest University, Winston-Salem, NC (PI: Kenneth Koch, MD)

The study will be implemented in two lead clinics (Wake Forest University and the University of Michigan) followed by the 5 additional clinical sites.

Patients with diabetic gastroparesis will be identified and registered for screening, CGMS training, and the run-in period at the clinical centers subject to the inclusion and exclusion criteria listed below. Eligible patients will be enrolled after completion of all required study procedures including successful learning of CGMS to inform insulin pump therapy, keying of all required data elements, and passing eligibility checks for the GLUMIT-DG study. Patients may be recruited directly into the GLUMIT-DG study or may be recruited from the Gastroparesis Registry.

Each clinical center will develop a recruitment plan. These plans will vary from clinic to clinic depending on the available pools of patients with diabetic gastroparesis and local recruitment resources. Clinics will attempt to recruit sufficient numbers of minorities and males.

4.2. Inclusion criteria

Patients with mild to moderate symptoms of diabetic gastroparesis will be studied. Patients should not have idiopathic or post-surgical gastroparesis etiologies and must satisfy the following inclusion criteria:

1. Age 18 – 70 years old at registration
2 Type 1 or type 2 diabetes mellitus for at least 2 years
3. Symptoms of gastroparesis (nausea, vomiting, early satiety, bloating fullness, discomfort) for at least 1 year prior to registration with a Gastroparesis Cardinal Symptom Index (GCSI) score of ≥18
4. Delayed gastric emptying on gastric emptying scintigraphy within 1 year of registration, defined as greater than 60% retention at 2 hours or greater than 10% retention at 4 hours

GPCRC\GLUMIT-DG\Protocol\Manall_87
3:12 pm Wednesday, August 12, 2009/klc

5. Hemoglobin Alc of at least 8.0% at registration. Individuals already receiving diabetes therapy via an insulin pump will be eligible for participation if, in the opinion of investigators, he/she may acquire additional benefit from CGMS that might improve glycemic control
6. Normal upper endoscopy within 1 year of registration
7. No clinical or imaging evidence of obstruction
8. Successful use of the CGMS during the screening period
9. Access to a computer with internet capabilities.

4.3. Exclusion criteria

Patients who satisfy any of the following exclusion criteria will be ineligible for enrollment in the study:

1. Prior gastric surgery including fundoplication
2. Other systemic diseases potentially causative of gastrointestinal symptoms
3. Acute or chronic renal insufficiency with creatinine >1.5 mg/dL
4. Psychiatric disease or eating disorder
5. Contradiction to gastric emptying breath test such as a known allergy to egg, wheat, or algae
6. Pregnancy
7. Any other condition which in the opinion of the investigators would impede compliance or hinder completion of the study

4.4. Run-in phase

Prior to enrollment into the GLUMIT-DG study, patients will undergo up to 8 weeks of a run-in phase in which they will learn to operate an insulin pump coupled with CGMS. At the end of the run-in phase, patients must show competency with operating the insulin pump and CGMS to be eligible for participation in the GLUMIT-DG study. Specifically, a patient must be able to check blood glucose levels with the MiniLink™ REAL-Time Transmitter CGMS as well as with the traditional finger stick method at least 4 times daily. In addition, patients must be able to manage the Minimed Paradigm® Insulin Pump which includes making adjustments to insulin dosing according to standards provided by the GLUMIT-DG study physician and diabetes educator. Patients must also be able to electronically transfer data from the CGMS via their home computer to the GLUMIT-DG study staff. Once a patient has shown the ability to complete all of these tasks and has confirmed their consent to participate, they may enroll into the GLUMIT-DG study.

GPCRC\GLUMIT-DG\Protocol\Manall_87
3:12 pm Wednesday, August 12, 2009/klc

5. Protocol procedures

5.1. Visit schedule overview

The patient-related activities of the GLUMIT-DG study can be divided into 3 phases:

- Screening to determine eligibility and receive CGMS training with demonstration of proficiency with the use of CGMS (3 visits over a minimum of 2 weeks up to a maximum of 8 weeks for gastric related testing and CGMS training), and the patient will need to produce at least 216 hours of CGMS readings during a consecutive 2-week period
- Run-in with insulin pump training and with the use of CGMS to aid the adjustment of insulin administration with the pump (4 visits over a minimum of 2 weeks up to a maximum of 8 weeks)
- Treatment and follow-up (6 visits over 24 weeks)

The visit and data collection schedule described below in detail is summarized in Appendix 9.2.

5.2. Screening and baseline data collection overview

Many of the GLUMIT-DG participants will come from the Gastroparesis Registry or from the patient rosters of the study physicians. Patients who appear to be eligible after chart review and completion of standard of care tests and procedures for gastroparesis will be invited to undergo screening for the GLUMIT-DG study. Patients considered by the clinical center study physician as likely to be eligible for participation in the GLUMIT-DG study may be consented, registered, and screened at a visit that is part of the ongoing clinical care of the patient. Screening tests should be completed according to the standard of care as outlined in the Standard Operating Procedures IV: Standard of Care for Patients with Diabetic Gastroparesis.

As part of the screening process for the GLUMIT-DG study, the patient must have a standardized 4 hour scintigraphic evaluation of gastric emptying using a low fat Egg Beaters meal available for review by the study physician as outlined in the Standard Operating Procedures I: Clinical Center Operations. The standard of care gastric emptying scintigraphy may have been obtained at any time within 1 year prior to the registration date in GLUMIT-DG or may be performed during screening. Patients must also have a negative upper endoscopy within the last year to rule out other potential causes of symptoms such as mechanical obstruction, inflammatory or other structural lesions of the GI tract, or non-gastrointestinal causes. Recording of screening data on study forms may not start until the patient has signed the GLUMIT-DG consent statement. Screening and baseline data collection procedures will include review of the patient's medical chart and completion of questionnaires, physical examination, hemoglobin A1c testing, monitoring of blood glucose values, and study specific clinical procedures on patients. Data abstracted from a patient's chart may

include laboratory, endoscopy, radiology, and gastric emptying scintigraphy test results. Prior therapy for gastroparesis and diabetes will also be reviewed.

All participants who sign the consent statement will be registered in the GLUMIT-DG study. Each participant who is registered in the GLUMIT-DG study and who starts screening will be accounted for at the end of the screening phase, as either a screening success (enrollment) or a screening failure. A screening failure is defined as a participant who signed the consent form and was registered in the GLUMIT-DG study, but is found to be ineligible prior to enrollment. Screening failures include patients who meet medical eligibility criteria but failed the CGMS training or changed their mind and do not consent to enrollment into the study after careful consideration of the study demands on their personal time. The reason for screening failure will be recorded on the GLUMIT-DG enrollment form and keyed in the study database.

Screening and baseline data collection procedures will be conducted over three clinic visits. The goal of the first screening visit is to obtain consent, register the prospective participant for GLUMIT-DG, start recording screening data regarding the study inclusion and exclusion criteria on case report forms, and initiate CGMS training; the goal of the second and third screening visits is to complete collection of baseline data including gastric emptying breath test, EGG, and satiety tests on patients who appear to be eligible and complete CGMS training. This separation of procedures between three study visits is somewhat arbitrary and is provided as a guideline. Study screening procedures and data collection can be organized as appropriate at each clinical center. The procedures completed during the screening phase include:

Gastric emptying scintigraphy: Patients who have not had a standardized 4 hour gastric emptying scintigraphy within 1 year of registration or whose previous gastric emptying scintigraphy is of inadequate quality must have a standard of care gastric emptying scintigraphy as part of screening prior to enrollment. Instructions for the gastric emptying scintigraphy are found in the GLUMIT-DG Standard Operating Procedures I: Clinical Center Operations.

Upper endoscopy: Patients who have not had an upper endoscopy within 1 year of registration must have a standard of care upper endoscopy as part of screening prior to enrollment.

- **Screening visit 1:** The patient should be in a fasting state (no food or drink except up to 120 mL (4 oz) of water after midnight the night before) for this visit. The patient will sign the GLUMIT-DG consent at or prior to screening visit 1 and will undergo a history and physical examination to identify other illness and contraindications for participation. In addition, blood will be drawn to measure their hemoglobin Alc level. A patient's hemoglobin A1c level must be 8.0% or higher in order to be eligible for this study. Laboratory test results that need to be recorded from chart review or obtained as part of screening include: complete blood count (CBC): white blood cells, red blood cells, white blood cell differential, hemoglobin, and platelets; a comprehensive metabolic panel: sodium, potassium, chloride, carbon dioxide, calcium, glucose, blood urea nitrogen (BUN), creatinine, albumin, total protein, alanine aminotransferase (ALT), aspartate aminotransferase (AST),

GLUMIT-DG Protocol

and bilirubin; and thyroid stimulating hormone (TSH). The Patient Assessment of Upper Gastrointestinal Disorders - Quality of Life (PAGI-QOL) and the Patient Assessment of Upper Gastrointestinal Disorders - Symptom Severity Index (PAGI-SYM) questionnaires will also be completed as well as the Baseline Medical History and Physical Examination forms.

Continuous Glucose Monitoring System (CGMS) training: At a clinic visit as early as possible during the screening phase, patients will be seen by a diabetes educator to undergo training on measuring their blood glucose levels using the CGMS. The CGMS involves wearing a sensor device, which has a needle attached that is inserted under the abdominal skin close to the stomach wall to continuously measure blood glucose levels. The sensor is calibrated according to blood glucose results taken from finger sticks. Patients will measure their blood glucose levels using a standard finger stick method and will be required to do so 4 times daily using a One Touch© Ultra glucose meter. During this period, patients will continue to take their regular diabetes medications or insulin treatment including those who already wear an insulin pump. This CGMS data will not be used for adjustment of diabetes therapy.

- **Screening visit 2:** Patients will return to the clinical center in a fasting state (no food or drink except up to 120 mL (4 oz) of water after midnight the night before) on a separate day after the first screening visit to continue with study procedures and baseline data collection for gastric emptying breath testing, electrocardiogram (ECG), and autonomic function testing. If patients normally take insulin, they will be asked to take only half of their normal long-acting insulin. Patients will have 10 mL of blood drawn for plasma banking to measure inflammatory and immune markers or plasma proteins to ascertain the effects of controlling blood glucose during the GLUMIT-DG study. Patients will have their blood glucose level measured using a finger stick method to ensure their blood glucose is less than 270 mg/dL. Once confirmation is made that the patient's blood glucose level is less than 270 mg/dL, a gastric emptying breath test will be performed.

Baseline gastric emptying breath test: Gastric emptying will be performed using the ^{13}*C-Spirulina* Gastric Emptying Breath Test (^{13}C-*Sp*GEBT) manufactured by Advanced Breath Diagnostics for clinical research[15,16]. Advanced Breath Diagnostics provides the standardized meal as an oral pharmaceutical product in compliance with the FDA's Good Manufacturing Practices. The patient must fast overnight for the baseline GEBT measurements. Before the GEBT can begin, the patient provides a baseline fasting (pre-meal) breath sample. The patient is then administered a 230 kCal meal consisting of a standard ^{13}C-labeled egg component and 6 saltine crackers. Four ounces of water accompanies the meal. The patient is asked to consume the meal within 10 minutes. A timer is set upon completion of the GEBT meal. Breath samples are then collected at 45 minutes, 90 minutes, 120 minutes, 150 minutes, and 180 minutes post-meal time points. Breath samples will be sent to the Advanced Breath Diagnostics reference laboratory for analysis by stable isotope ratio

GLUMIT-DG Protocol

mass spectrometry. Detailed instructions for the GEBT can be found in the GLUMIT-DG Standard Operating Procedures I: Clinical Center Operations, section 6.4.

Autonomic function with ECG: Patients will have a baseline ECG followed by an ECG tracing for heart rate variability. Patients will be asked to remain in the supine position while breathing slowly for a five minute period. From this ECG tracing, vagal cholinergic function will be estimated by quantifying changes in heart rate during inspiration and respiration. R-R interval will be analyzed for mid and high frequency fluctuations (max to min ratio normal is 30:150).

- **Screening visit 3:** Patients will return to the clinical center in a fasting state (no food or drink except up to 120 mL (4 oz) of water after midnight the night before) at least one day after the second screening visit has taken place. If patients normally take insulin, they will be asked to take only half of their normal long-acting insulin. Patients will also have their blood glucose levels measured using the finger stick method to ensure their blood glucose is less than 270 mg/dL. Once confirmation is made that the patient's blood glucose level is less than 270 mg/dL, the electrogastrograms with non-caloric and caloric satiety tests will be performed.

Electrogastrogram (EGG) with satiety testing: Patients may adjust their insulin and take their normal morning medications with a small amount of water (up to 120 mL) up to two hours prior to the EGG with satiety testing, but should refrain from coffee, juice, or tea. EGG is the recording of the myoelectrical activity of the stomach using electrodes similar to an electrocardiogram. EGG electrodes are placed on the abdominal skin. Skin preparation for these electrodes will consist of cleaning the skin with an electrode paste and then applying a conduction gel which is then wiped off. If needed, the abdominal skin where electrodes will be positioned is shaved.

Non-caloric satiety testing: For the non-caloric water load satiety test, patients will undergo a 15 minute fasting baseline EGG recording and a 30 minute post-water load EGG recording. After completion of the baseline period, patients will drink bottled spring water refrigerated at 4 degrees C for a 5 minute period until they feel completely full. Patients will drink from an opaque 150 mL cup which will be filled out of sight so that the volume consumed is not readily apparent to minimize the potential of introducing bias. In addition, no information regarding the total volume of water ingested will be communicated to the patient. The total volume of water ingested will be recorded on the EGG and Water Load Satiety Test form. Symptoms of nausea, bloating, abdominal discomfort, hunger, and stomach fullness will be recorded on a visual analog scale at baseline and at 10, 20, and 30 minutes after ingestion of the cool water. The EGG will be recorded and analyzed for the 15 minute fasting baseline and post water-load period at 0-10, 11-20 and 21-30 minutes after ingestion of the water load.

GLUMIT-DG Protocol

Caloric satiety testing: Approximately two hours after completion of the non-caloric water load test with EGG recording, patients will undergo a caloric satiety test. Patients will undergo a 15 minute baseline fasting and 60 minute post-nutrient EGG recording. After completion of the baseline period, patients will drink a cup (150 mL) of refrigerated (at 4 degrees C) Ensure® (1.1 kcal/mL) at a rate of 150 mL every 5 minutes until they feel completely full. Patients will drink from an opaque cup which will be filled out of sight so that the volume consumed is not readily apparent to minimize the potential of introducing an assessment bias. In addition, no information regarding the total volume of Ensure® ingested will be communicated to the patient. The total volume of Ensure® ingested will be recorded on the EGG and Satiety Test form. The satiety test will be analyzed by the total amount of Ensure® consumed. Symptoms of nausea, bloating, abdominal discomfort, hunger, and stomach fullness will be recorded on a visual analog scale at baseline and at 10, 20, 30, and 60 minutes after ingestion of Ensure®. The electrodes will then be removed. The EGG will be analyzed for the fasting and post nutrient load periods corresponding to symptom assessments at 0-10, 11-20, 21-30, 31-40, 41-50 and 51-60 minute intervals. The percentage distribution of the power in the normal frequency range (2.5-3.75 cycles per minute [cpm]), tachygastria (>3.75-10 cpm), bradygastria (<2.5 cpm) and duodenal-respiration (>10 cpm) will be calculated. A power ratio comparing mean signal amplitudes between postprandial and preprandial power will be calculated. The dominant frequency, and the percentage of time in the dominant frequency will be calculated and recorded.

Patients will receive 1 unit of insulin for every 15 grams of Ensure® consumed during the caloric satiety testing to reduce the likelihood of severe postprandial hyperglycemia. Patients blood glucose levels will be closely monitored using the CGMS or with the finger stick method, as needed. Patients will be monitored for 2 hours after beginning the Ensure® satiety testing to assure their blood glucose level does not exceed 300 mg/dL. If a patient’s blood glucose level exceeds 300 mg/dL, additional insulin will be given to bring it down to 250 mg/dL.

At the conclusion of the EGG with satiety testing, patients will be seen again by the diabetes educator to continue with their CGMS training. If further training is needed, per the judgment of the diabetes educator or the diabetologist, extra visits or phone calls may be scheduled. Once the training is completed (as per the diabetes educator or the diabetologist), the patient will be requested to use the CGMS for a period of 2 weeks, perform a weekly uploading of the data to the Carelink web page and perform 4 finger sticks per day which will be used for calibration of the CGMS. If 216 hours of good CGMS data is obtained during the 2-week period, training will be considered successful and the patient will proceed with the run-in period. Alternatively, if a patient has not demonstrated adherence with checking blood glucose levels using the One Touch© Ultra glucose meter 4 times daily, or if the CGMS data do not fulfill the criteria of 216 hours of good data, the patient will be provided additional instruction and will be allowed to repeat the CGMS proficiency

test for an additional 2-week period. If the patient is unsuccessful after the second trial, he/she will be considered ineligible for the GLUMIT-DG study.

5.3. Run-in visits with insulin pump training

After successful completion of CGMS training, patients will return for four weekly visits, for a minimum of two weeks up to an 8-week learning period to receive training on the usage of an insulin pump coupled with CGMS. Participants will be trained on how to use a computer to send the data gathered by the CGMS and insulin pump to their study physician and diabetes educator. During the run-in period insulin pump infusion parameters will be adjusted to optimize regulation of blood glucose levels, using information from both the CGMS and the finger sticks.

- **Run-in visit 1**: Participants will receive instruction on use of the real time CGMS, including the uploading of the data using the CareLink system as well as instructions on glycemia management. The CGMS has a feature known as “REAL-Time Alarms” which will be set to warn patients when their glucose levels are very low or very high. The hypoglycemic alarm threshold will be set at 80 mg/dL and the hyperglycemic alarm threshold will be set at 240 mg/dL. The high and low snooze alarms will be set between 20-30 minutes to alert patients during this time increment whenever hyperglycemia or hypoglycemia episodes are forecasted to occur.

- **Run-in visit 2**: This visit should be planned within a week after the first run-in visit. Patients will be taught insulin infusion pump mechanics and will be started on an insulin pump filled with saline. The purpose of filling the pump with saline is to ensure that the patient can tolerate wearing it before it is filled with insulin.

- **Run-in visit 3**: Patients will stop previous diabetes treatment and insulin administration with the pump will be initiated according to predetermined insulin dose parameters. For those patients who entered the study using an insulin pump, parameters will be adjusted as per CGMS readings. Carbohydrate to insulin ratios, insulin sensitivity, insulin on board (3 to 5 hours), and glycemic targets will be established by the study staff. Patients will be instructed to only use the bolus wizard feature of the pump when delivering insulin doses for meals to ensure the proper amount of insulin is delivered. Due to the presence of gastroparesis, meal boluses for control of postprandial hyperglycemia will be started when the CGMS confirms that glucose concentration starts to rise after meal ingestion. This approach is aimed at minimizing a mismatch in insulin bolus administration and nutrient absorption due to delayed and possibly erratic gastric emptying. In addition to intensive education on glycemic control, participants will be instructed in depth on symptoms and signs of hypoglycemia and steps to correct it without caloric overreplacement to avoid rebound hyperglycemia.

GLUMIT-DG Protocol

• **Run-in visit 4**: Patients will review their real time CGMS results and receive additional education on glycemia management. They will also be taught how to use glucose trends as displayed by the CGMS to modify their insulin doses and to ingest carbohydrates if the CGMS identifies a falling level of glucose, which may lead to hypoglycemia.

In order for a patient to successfully complete the run-in period; that may span up to eight weeks before enrollment, a thorough understanding of operating both the CGMS and insulin pump is necessary. Patients will competently know how to upload the CGMS data from a computer to a personalized web page using the CareLink system. Patients will understand the mechanics of the hypoglycemic alarms and how to prevent “insulin stacking”. Management of blood glucose, especially to prevent hypoglycemic and hyperglycemic events is necessary. An understanding of using glucose trends to modify insulin doses will also be expected. At the end of the run-in period, each patient must be able to complete all of these tasks before enrollment into the study.

5.4. Enrollment

Enrollment into the GLUMIT-DG study may occur once a patient has met all the eligibility criteria and has shown the ability to master insulin pump therapy and glucose monitoring with CGMS. Specifically, a patient must be able to check blood glucose levels with the MiniLink™ REAL-Time Transmitter CGMS as well as with the traditional finger stick method at least 4 times daily. In addition, patients must be able to manage the Minimed Paradigm® Insulin Pump which includes making adjustments to insulin dosing according to instructions provided by the GLUMIT-DG study physician and diabetes educator. Patients must also be able to transfer data from the CGMS via their home computer to the GLUMIT-DG study staff. Once a patient has shown the ability to complete all of these tasks and has confirmed consent to participate, he/she may enroll into the GLUMIT-DG study.

5.5. Follow-up visits

Patients will return for follow-up visits, once every 4 weeks over a 24-week period after enrollment. During these visits, patients will meet with the diabetes educator and have their insulin pump parameters (basal rate, carbohydrate to insulin ratio for meals, etc.) adjusted according to blood glucose readings from both the CGMS and finger sticks.

The blood glucose measurements of patients will be reviewed by the study staff on a weekly basis using the CareLink system and suggestions for insulin pump adjustments will be explained to the patient. In addition, symptoms of gastroparesis will be managed according to the prevailing standard of care including appropriate use of antiemetic and prokinetic medications as outlined in SOP IV: Standard of Care for Patients with Diabetic Gastroparesis.

GLUMIT-DG Protocol

Insulin dose adjustments

Basal rate

The basal rate will be adjusted in increments or decrements of 0.05 to 0.2 units of insulin per hour based on the non-meal glycemia (with special attention to the overnight glycemia) as per the glucose data gathered by the CGMS as well as the finger stick glycemia.

MEAL BOLUSES

The adjustment for meal related glycemia is of special interest in the GLUMIT-DG study since gastric emptying may be erratic and delayed. For the GLUMIT-DG study, the following guidelines will be used for adjustment of meal boluses at all subsequent follow-up visits or phone contacts.

Insulin administered for meal(s) will be tailored using a dual wave pattern. The amount of insulin for the specific meal will be estimated using the carbohydrate to insulin ratio (CHO:I). This ratio is the estimated amount of meal carbohydrate that one unit of insulin metabolizes. This ratio is calculated initially by the following formulas (see also Section 3.3):

(1) 450/(BW x 0.84) for Type 2 diabetes, and

(2) 450/(BW x 0.2) for Type 1 diabetes

The carbohydrate to insulin ratio is in the following units: grams of meal carbohydrate for every unit of insulin. The ratio is entered into the insulin pump. When the patient is about to eat or has already eaten, he/she enters into the pump the estimated amount of carbohydrate ingested during the meal or the meal the patient is preparing to eat. The microchip in the pump uses the pre-entered CHO:I ratio to calculate the bolus of insulin to be delivered. For example, if the meal carbohydrate is calculated as 90 grams and the CHO:I ratio is 30, then the pump will calculate a total of 3 units of insulin. If the same amount of meal carbohydrate is eaten and the CHO:I ratio is 10, then the total amount of meal insulin calculated is 9 units. Thus a higher CHO:I ratio yields a lower calculated insulin bolus and vice versa. The pump will prompt the user to confirm acceptance of the suggested dose and will not deliver the bolus unless the patient accepts the suggestion from the pump.

The bolus of insulin can be delivered in TOTAL during a short period of time (square wave) or it can be distributed to be delivered as a prolonged wave. Moreover, the latter can be configured by delivering a certain % of the dose within the first hour after meal ingestion and the rest during hours 2 and 3 after meal ingestion (extended bolus). These times can be tailored more as per the judgement

GLUMIT-DG Protocol

of the diabetologist and diabetes educator. For obvious reasons, the second approach is better suited for patients with gastroparesis since it diminishes the possibility of hypoglycemia and will be used in the GLUMIT-DG study.

For adjustment of the meal boluses, an approach to adjust the total amount of insulin in the bolus by changes in the CHO:I ratio (an increase in CHO:I ratio decreases insulin dose, a decrease in CHO:I ratio increases insulin dose) will be used. The specific insulin dose titration algorithm is in the table below.

Carbohydrate to Insulin Dose Titration Algorithm Programmed in the Insulin Pump

1 hour glucose mg/dL	3 hour glucose mg/dL	Suggested change in CHO:I ratio*	Suggested change in % bolus in first hour (relative to extended bolus)**	ROW
<80	<80	Increase by 2		1
<80	80-180	Increase by 1		2
<80	180-250		Decrease by 10%	3
<80	>250		Decrease by 20%	4
**80-180**	**80-180**			5
80-180	180-250	Decrease by 1		6
80-180	>250	Decrease by 2		7
180-250	<80		Increase by 20%	8
180-250	80-180		Increase by 10%	9
180-250	180-250	Decrease by 1		10
180-250	>250	Decrease by 2	Decrease by 10%	11
>250	<80		Increase by 20%	12
>250	80-180		Increase by 10%	13
>250	180-250	Decrease by 1	Increase by 10%	14
>250	>250	Decrease by 2		15

* CHO:I ratio = grams of carbohydrate in the meal: per one unit of insulin (this ratio estimates the amount of carbohydrate in grams that are metabolized by 1 unit of insulin)

** The % bolus is the amount of meal insulin delivered in the first hour after initiation of bolus administration; extended is the amount of insulin delivered in the subsequent 2-4 hours.

GLUMIT-DG Protocol

Columns 1 and 2 in the table indicate the glycemia at time = 1 hour (column 1) and time = 3 hours (column 2) AFTER meal ingestion. Column 3 indicates the suggested change in carbohydrate:insulin ratio (CHO:I). As can be seen from the Table, when glucose values are low, then the CHO:I ratio is suggested to be increased (see row #1 as specific example) to decrease the total amount of meal insulin delivered in subsequent meals. Contrary to the latter, the CHO:I needs to be decreased if the glucose is high (see last row for specific example). Since the process is cumulative, if the glucose levels are not corrected with an initial change in the CHO:I ratio, then it can be adjusted again when new glucose levels are available.

The distribution of the insulin bolus (as a percent distribution for the post meal period) will also be adjusted based on the finger stick at hour = 1 and hour = 3. The actual change in percentage itself will NOT affect the total amount of insulin of the bolus, only its distribution in time. The suggested adjustments are listed in column 4 of the Table. A decrease in the percentage will result in a net decrease in the first hour and an increase in the extended period. This is the case for example in row 3 of the table. The glucose at time = 1 hour is low (less than 80 mg/dL) but is above target at time = 3 hour. By decreasing the percent bolus to 10% in the first hour, less insulin is delivered in the first hour and the same amount of insulin is shifted into the extended bolus. An example of the contrary is in row 8 where 20% increase in the percent bolus shifts insulin into the first hour (finger stick = 180-250 mg/dL) and decreases this amount in the extended bolus (since finger stick is <80 mg/dL).

There are specific combinations of glucose readings that will trigger a change in TOTAL insulin as well as in its distribution in time, for example row 11.

Some special precautions will be built into bolus administration for meals as follows:

1. Assuming that gastric emptying (hence GI absorption of nutrients) is highly unpredictable, the bolus administration will not start until the CGMS stream of data indicates that blood glucose has started to rise (as a proxy for GI absorption).

2. The shape of the bolus will be adjusted based on the glycemic pattern observed after meals; we anticipate that we will need to use dual-wave boluses or a combination of dual-wave boluses and complementary temporary basal rates. It is safe to assume that quite a bit will be learned during the CGMS training and run-in period and that the profiles of glycemia will be highly individualized. Thus, we will use CGMS data to predict the best shape of meal boluses.

- **Week 4 visit:** Patients will meet with a diabetes educator to review blood glucose levels and to make necessary adjustments to insulin doses through the insulin pump. The Follow-up Medical History form will be completed. Any unanticipated adverse events will be reviewed with the patient and recorded on case report forms.

GLUMIT-DG Protocol

- **Week 8 visit:** Patients will meet with a diabetes educator to review blood glucose levels and to make necessary adjustments to insulin administration by adjustment of parameters of the pump. The Follow-up Medical History form will be completed. Any unanticipated adverse events will be reviewed with the patient and recorded on case report forms.

- **Week 12 (Day 1 visit):** Patients will complete this follow-up study visit over two days. On the first day, patients should be in a fasting state (no food or drink except up to 120 mL of water after midnight the night before). Patients will also be instructed to cut back the amount of insulin they receive from their insulin pump by half before arriving for their appointment. On arriving at the clinic, the patient's blood glucose level will be checked to ensure it is less than 270 mg/dL. If the patient's blood glucose level is above 270 mg/dL, GEBT will be rescheduled to be done within one week. Medical history will be obtained including a physical exam and blood will be drawn for hemoglobin A1c, complete blood count, and a comprehensive metabolic panel: sodium, potassium, chloride, carbon dioxide, calcium, glucose, blood urea nitrogen (BUN), creatinine, albumin, total protein, alanine aminotransferase (ALT), aspartate aminotransferase (AST), and bilirubin. Patients will also have 10 mL of blood drawn for plasma banking to measure inflammatory and immune markers, or plasma proteins to ascertain the effects of controlling blood glucose. Gastric emptying breath testing (GEBT) and autonomic function with ECG will take place. Patients will also complete the Patient Assessment of Upper Gastrointestinal Disorders-Symptom Severity Index (PAGI-SYM) and the Patient Assessment of Upper Gastrointestinal Disorders-Quality of Life (PAGI-QOL) questionnaires. Any unanticipated adverse events will be reviewed with the patient and recorded on case report forms.

Follow-up gastric emptying breath test: The patient must fast overnight for the baseline GEBT measurements. Before the GEBT can begin, the patient provides a baseline fasting (pre-meal) breath sample. The patient is then administered a 230 kCal meal consisting of a standard ^{13}C-labeled egg component and 6 saltine crackers. Four ounces of water accompanies the meal. The patient is asked to consume the meal within 10 minutes. A timer is set upon completion of the GEBT meal. Breath samples are then collected at 45 minutes, 90 minutes, 120 minutes, 150 minutes, and 180 minutes post-meal time points. Breath samples will be sent to the Advanced Breath Diagnostics reference laboratory for analysis by stable isotope ratio mass spectrometry. Further instructions for the GEBT can be found in the GLUMIT-DG Standard Operating Procedures I: Clinical Center Operations.

Autonomic function with ECG: Patients will have a baseline ECG done followed by an ECG tracing for heart rate variability. Patients will be requested to remain in the supine position while breathing slowly for a five minute period. From this ECG tracing, vagal cholinergic function will be estimated by quantifying changes in heart rate during inspiration and respiration. R-R interval will be analyzed for mid and high frequency fluctuations (max to min ratio normal is 30:150).

GLUMIT-DG Protocol

- **Week 12 (Day 2 visit):** Patients will return to the clinical center in a fasting state (no food or drink except up to 120 mL of water after midnight the night before) at least one day after the first week 12 follow-up visit, preferably the next day. Patients will be asked to take only half of their normal insulin. Patients will have their blood glucose level measured using the finger stick method to ensure their blood glucose level is less than 270 mg/dL. Once confirmation is made that the patient's blood glucose level is less than 270 mg/dL, electrogastrography (EGG) with satiety testing will be performed.

Electrogastrogram (EGG) with satiety testing: Patients will adjust their insulin and take their normal morning medications with a small amount of water (up to 120 mL) up to two hours prior to the EGG with satiety testing, but should refrain from coffee, juice, or tea. Electrogastrogram electrodes are placed on the abdominal skin. Skin preparation for these electrodes will consist of cleaning the skin with an electrode paste and then applying a conduction gel which is then wiped off. If needed, the abdominal surface where electrodes will be positioned is shaved.

Non-caloric satiety testing: For the non-caloric water load satiety test, patients will undergo a 15 minute fasting baseline EGG recording and a 30 minute post-water load EGG recording. After completion of the baseline period, patients will drink bottled spring water refrigerated at 4 degrees C for a 5 minute period until they feel completely full. Patients will drink from an opaque 150 mL cup which will be filled out of sight so that the volume consumed is not readily apparent to minimize the potential of introducing bias. In addition, no information regarding the total volume of water ingested will be communicated to the patient. The total volume of water ingested will be recorded on the EGG and Water Load Satiety Test form. Symptoms of nausea, bloating, abdominal discomfort, hunger, and stomach fullness will be recorded on a visual analog scale at baseline and at 10, 20, and 30 minutes after ingestion of the cool water. The EGG will be recorded and analyzed for the 15 minute fasting baseline and post water-load period at 0-10, 11-20 and 21-30 minutes after ingestion of the water load.

Caloric satiety testing: Approximately two hours after completion of the non-caloric water load test with EGG recording, patients will undergo a caloric satiety test. Patients will undergo a 15 minute baseline fasting and 60 minute post-nutrient EGG recording. After completion of the baseline period, patients will drink a cup (150 mL) of refrigerated (at 4 degrees C) Ensure® (1.1 kcal/mL) at a rate of 150 mL every 5 minutes until they feel completely full. Patients will drink from an opaque cup which will be filled out of sight so that the volume consumed is not readily apparent to minimize the potential of introducing an assessment bias. In addition, no information regarding the total volume of Ensure® ingested will be communicated to the patient. The total volume of Ensure® ingested will be recorded on the EGG and Satiety Test form. The satiety test will be analyzed by the total amount of Ensure® consumed. Symptoms of nausea, bloating, abdominal discomfort, hunger, and stomach fullness will be recorded on a visual analog scale at baseline and at

GLUMIT-DG Protocol

10, 20, 30, and 60 minutes after ingestion of Ensure®. The electrodes will then be removed. The EGG will be analyzed for the fasting and post nutrient load periods corresponding to symptom assessments at 0-10, 11-20, 21-30, 31-40, 41-50 and 51-60 minute intervals. The percentage distribution of the power in the normal frequency range (2.5-3.75 cycles per minute [cpm]), tachygastria (>3.75-10.0 cpm), bradygastria (<2.5 cpm) and duodenal-respiration (>10 cpm) will be calculated. A power ratio comparing mean signal amplitudes between postprandial and preprandial power will be calculated. The dominant frequency, and the percentage of time in the dominant frequency will be calculated and recorded.

Patients will receive 1 unit of insulin for every 15 grams of Ensure® consumed during the caloric satiety testing to reduce the likelihood of severe postprandial hyperglycemia. Patients blood glucose levels will be closely monitored using the CGMS or with the finger stick method, as needed. Patients will be monitored for 2 hours after beginning the Ensure® satiety testing to assure their blood glucose level does not exceed 300 mg/dL. If a patient's blood glucose level exceeds 300 mg/dL, additional insulin will be given to bring it down to 250 mg/dL.

- **Week 16 visit:** Patients will meet with a diabetes educator to review blood glucose levels and to make necessary adjustments to insulin doses by changing parameters of the insulin pump. The Follow-up Medical History form will be completed. Any unanticipated adverse events will be reviewed with the patient and recorded on case report forms.

- **Week 20 visit:** Patients will meet with a diabetes educator to review blood glucose levels and to make necessary adjustments to insulin doses by changing of parameters of the insulin pump. The Follow-up Medical History form will be completed. Any unanticipated adverse events will be reviewed with the patient and recorded on case report forms.

GLUMIT-DG Protocol

- **Week 24 (Day 1 visit):** Patients will complete this visit over two days. On the first day, patients should be in a fasting state (no food or drink except up to 120 mL of water after midnight the night before). Patients will also be instructed to cut back the amount of insulin they receive from their insulin pump by half before arriving for their appointment. On arriving at the clinic, the patient blood glucose levels will be checked to ensure it is less than 270 mg/dL. If the patient's blood glucose level is above 270 mg/dL, gastric emptying breath testing will be rescheduled to be done within one week. Medical history will be obtained including a physical exam and blood will be drawn for hemoglobin A1c, complete blood count, and a comprehensive metabolic panel: sodium, potassium, chloride, carbon dioxide, calcium, glucose, blood urea nitrogen (BUN), creatinine, albumin, total protein, alanine aminotransferase (ALT), aspartate aminotransferase (AST), and bilirubin. Patients will also have 10 mL of blood drawn for plasma banking to measure inflammatory and immune markers or plasma proteins to ascertain the effects of controlling blood glucose. Gastric emptying breath testing (GEBT) and autonomic function with ECG testing will take place. Patients will also complete the Patient Assessment of Upper Gastrointestinal Disorders-Symptom Severity Index (PAGI-SYM) and the Patient Assessment of Upper Gastrointestinal Disorders-Quality of Life (PAGI-QOL) questionnaires. Any unanticipated adverse events will be reviewed with the patient and recorded on case report forms.

Follow-up gastric emptying breath test: Before the GEBT can begin, the patient provides a baseline fasting (pre-meal) breath sample. The patient is then administered a 230 kCal meal consisting of a standard ^{13}C-labeled egg component and 6 saltine crackers. Four ounces of water accompanies the meal. The patient is asked to consume the meal within 10 minutes. A timer is set upon completion of the GEBT meal. Breath samples are then collected at 45 minutes, 90 minutes, 120 minutes, 150 minutes, and 180 minutes post-meal time points. Breath samples will be sent to the Advanced Breath Diagnostics reference laboratory for analysis by stable isotope ratio mass spectrometry. Further instructions for the GEBT can be found in the GLUMIT-DG Standard Operating Procedures I: Clinical Center Operations. An electrocardiogram (ECG) and autonomic function testing will also take place during this visit.

Autonomic function with ECG: Patients will have a baseline ECG done followed by an ECG tracing for heart rate variability. Patients will be requested to remain in the supine position while breathing slowly for a five or six minute period. From this ECG tracing, vagal cholinergic function will be estimated by quantifying changes in heart rate during inspiration and respiration. R-R interval will be analyzed for mid and high frequency fluctuations (max to min ratio normal is 30:150).

- **Week 24 (Day 2 visit):** Patients will return to the clinical center in a fasting state (no food or drink except up to 120 mL of water after midnight the night before) at least one day after the first week 12 follow up visit, preferably the next day. Patients will be asked to take only half of their normal long-acting insulin. Patients will have their blood glucose level measured using the finger stick method to ensure their blood glucose level is less than 270 mg/dL. Once confirmation is made

GLUMIT-DG Protocol

that the patient's blood glucose level is less than 270 mg/dL, electrogastrography (EGG) with satiety test will be performed.

Electrogastrogram (EGG) with satiety testing: Patients will adjust their insulin and take their normal morning medications with a small amount of water (up to 120 mL) up to two hours prior to the EGG with satiety testing measurement, but should refrain from coffee, juice, or tea. EGG electrodes are placed on the abdominal skin. Skin preparation for these electrodes will consist of cleaning the skin with an electrode paste and then applying a conduction gel which is then wiped off. If needed, the abdominal surface where electrodes will be positioned is shaved.

Non-caloric satiety testing: For the non-caloric water load satiety test, patients will undergo a 15 minute fasting baseline EGG recording and a 30 minute post-water load EGG recording. After completion of the baseline period, patients will drink bottled spring water refrigerated at 4 degrees C for a 5 minute period until they feel completely full. Patients will drink from an opaque 150 mL cup which will be filled out of sight so that the volume consumed is not readily apparent to minimize the potential of introducing bias. In addition, no information regarding the total volume of water ingested will be communicated to the patient. The total volume of water ingested will be recorded on the EGG and Water Load Satiety Test form. Symptoms of nausea, bloating, abdominal discomfort, hunger, and stomach fullness will be recorded on a visual analog scale at baseline and at 10, 20, and 30 minutes after ingestion of the cool water. The EGG will be recorded and analyzed for the 15 minute fasting baseline and post water-load period at 0-10, 11-20 and 21-30 minutes after ingestion of the water load.

Caloric satiety testing: Approximately two hours after completion of the non-caloric water load test with EGG recording, patients will undergo a caloric satiety test. Patients will undergo a 15 minute baseline fasting and 60 minute post-nutrient EGG recording. After completion of the baseline period, patients will drink a cup of (150 mL) refrigerated (at 4 degrees C) Ensure® (1.1 kcal/mL) at a rate of 150 mL every 5 minutes until they feel completely full. Patients will drink from an opaque cup which will be filled out of sight so that the volume consumed is not readily apparent to minimize the potential of introducing an assessment bias. In addition, no information regarding the total volume of Ensure® ingested will be communicated to the patient. The total volume of Ensure® ingested will be recorded on the EGG and Satiety Test form. The satiety test will be analyzed by the total amount of Ensure® consumed. Symptoms of nausea, bloating, abdominal discomfort, hunger, and stomach fullness will be recorded on a visual analog scale at baseline and at 10, 20, 30, and 60 minutes after ingestion of Ensure®. The electrodes will then be removed. The EGG will be analyzed for the fasting and post nutrient load periods corresponding to symptom assessments at 0-10, 11-20, 21-30, 31-40, 41-50 and 51-60 minute intervals. The percentage distribution of the power in the normal frequency range (2.5-3.75 cycles per minute [cpm]), tachygastria (>3.75- 10.0 cpm), bradygastria (<2.5 cpm) and duodenal-respiration (>10 cpm) will be calculated. A power ratio comparing mean signal amplitudes between postprandial and preprandial

power will be calculated. The dominant frequency, and the percentage of time in the dominant frequency will be calculated and recorded.

Patients will receive 1 unit of insulin for every 15 grams of Ensure® consumed during the caloric satiety testing to reduce the likelihood of severe postprandial hyperglycemia. Patients blood glucose levels will be closely monitored using the CGMS or with the finger stick method, as needed. Patients will be monitored for 2 hours after beginning the Ensure® satiety testing to assure their blood glucose level does not exceed 300 mg/dL. If a patient's blood glucose level exceeds 300 mg/dL, additional insulin will be given to bring it down to 250 mg/dL.

5.6. Standardized questionnaires

Two standardized questionnaires will be administered to patients enrolled in the GLUMIT-DG study. Questionnaires will be administered at baseline (prior to enrollment) and during follow-up at specified intervals (see Appendix 9.2 for the data collection schedule). The purpose of the questionnaires is to obtain important information regarding gastroparesis symptoms and health-related quality of life.

Patient Assessment of Upper Gastrointestinal Disorders - Symptom Severity Index (PAGI-SYM). A validated 20 question questionnaire that measures symptoms of gastroparesis, dyspepsia, and gastroesophageal reflux disease on a 0 to 5 scale for each symptom [17].

Patient Assessment of Upper Gastrointestinal Disorders - Quality of Life (PAGI-QOL). A validated 30 question questionnaire that measures quality of life in upper gastrointestinal disorders [18].

5.7. Specimen repository

Ten milliliters of blood will be collected for plasma separation and banking at screening and at follow-up 12 and 24 week visits. Plasma will be separated and stored for use as approved by the Steering Committee of the GpCRC (see Appendix 9.3 for whole blood draw schedule). The blood will be drawn in the morning during the study visit while the patient is still fasting. The blood will be separated into plasma and will be divided into 0.5 mL aliquots. Plasma aliquots will be kept in a freezer at -70 degrees C and will be sent to the NIDDK Biosample Repository for banking. The plasma will be analyzed for inflammatory, immune markers, or plasma proteins for the effects controlling blood glucose has on disease biomarkers.

6. Safety monitoring

6.1. Safety issues

Safety issues can be divided into (a) safety concerns related to insulin pump therapy, (b) safety issues related to CGMS, (c) safety concerns related to management of hypoglycemic and hyperglycemic episodes, (d) safety concerns related to study procedures, and (e) concerns related to patient privacy. The following paragraphs discuss the important potential adverse events and the proposed safeguards to minimize the risks involved by participating in the GLUMIT-DG study.

Safety concerns related to insulin pump therapy and CGM: Patients in this study will use the One TouchⒸ finger stick meter as well as the CGMS MiniLink™ REAL-Time Transmitter. Because these devices will be inserted subcutaneously, there is a risk for the patient to develop an infection. The two plastic needles that will be used for the CGMS and the insulin pump also carry an infection risk. However, with proper technique and good care, the infection risk is small. If patients notice any discoloration of the skin or signs of inflammation (redness, swelling, pain), they will be required to contact the study investigator immediately. Patients may need treatment with antibiotics, if indicated. The needles may also cause hematomas (the needles may pierce small vessels and blood may leak). Hematomas return to normal unless the areas become infected. Very rarely, some people are allergic to the glue or the material used to secure the plastic needle to the skin; there may also be itching, redness and swelling. If this reaction occurs, medication administration may be necessary.

Safety concerns related to incidence and management of hypoglycemic and hyperglycemic episodes: Diabetic patients with gastroparesis run the risk of developing hypoglycemic or hyperglycemic episodes. In the event that a hypoglycemic or hyperglycemic episode should occur, appropriate treatment measures will be taken to resolve the event. A description of the treatment for hypoglycemic and hyperglycemic events during participation in the GLUMIT-DG study is as follows:

HYPOGLYCEMIA

Mild hypoglycemic episodes

For a **hypoglycemic event** to be considered mild, the capillary glucose will be 50 - 70 mg/dL as revealed on the CGMS and corroborated by a finger stick reading; and the patient is fully capable of self treatment without the need of third party assistance. Mild hypoglycemia may be symptomatic or asymptomatic. The following treatment protocol for mild hypoglycemic episodes will be followed:

1. If the glucose value is 50 - 70 mg/dL, it should be treated by ingestion of 15-20 grams of carbohydrates (e.g., 4-5 glucose tablets, 5-6 Lifesavers, 4-6 oz. of a regular [non diet] soft drink, or 8 oz. low fat milk, 4 teaspoons of sugar dissolved in a liquid or juice).
2. Blood glucose should be self-tested 15-20 minutes after ingestion of carbohydrates and therapy will be repeated as above, as needed. Cycle should be repeated until glucose level is 80 mg/dL or above.
3. Once episode is resolved, if no meal is scheduled the next 2 hours, a mixed nutrient snack, including a carbohydrate, protein, and fat will be ingested right after the initial therapy and after resolution of episode to prevent another episode.

Moderate hypoglycemic episode

For a **hypoglycemic event** to be considered moderate, the capillary glucose will be below 50 mg/dL as revealed on the CGMS and corroborated by a finger stick reading; and the patient is fully capable of self-treatment, without the need of third party assistance. The following protocol for treatment of **moderate hypoglycemia** will be followed:

1. If blood glucose level is less than 50 mg/dL, the patient should ingest 30 grams of carbohydrates (e.g., 6-8 glucose tablets).
2. Blood glucose will be tested 15-20 minutes after ingestion of carbohydrate and therapy will be repeated as above, as needed, and repeated until glucose level is 80 mg/dL or above.
3. Once the episode is resolved, if a meal is not scheduled within the next 2 hours, a mixed nutrient snack, including carbohydrate, protein, and fat should be ingested right after resolution of episode to prevent another episode.

Severe hypoglycemic episodes

If the capillary glucose level is below 50 mg/dL, as revealed on the CGMS and corroborated by a finger stick reading; and the patient is incapable of self-treatment, hence in need of assistance from a third party (friend, relative, paramedic), the following treatment protocol will be followed:

<u>For patients that are conscious and cooperative:</u>

1. Turn patient on side and administer 1 mg of glucagon (intramuscular, subcutaneous, or intravenous) using the provided kit.
2. Check finger stick glucose value in 10 minutes.
3. If consciousness and cooperation are fully restored and finger stick glucose value is above 80 mg/dL, provide with 20 grams of sugar (e.g., 4-5 glucose tablets, 5-6 Lifesavers, 4-6 oz. of a regular [non diet] soft drink, or 8 oz. low fat milk, 4 teaspoons of sugar dissolved in a liquid or juice) and check glucose level again in 20 minutes.

4. If finger stick glucose value is partially restored >60-80 mg/dL but patient remains conscious and fully cooperative, provide patient with another 20 grams of carbohydrate and repeat cycle until finger stick glucose value is >80 mg/dL and stable.
5. If finger stick glucose value is NOT above 60 mg/dL at the 10 minute check, call 911. If patient is still cooperative, offer 20 grams of carbohydrate; repeat cycle until paramedics arrive.

For patients that are unconscious or neurologically impaired:

1. Turn patient on side and administer 1 mg of glucagon (intramuscular, subcutaneous, or intravenous) using the provided kit.
2. Call paramedics or 911 (if haven't already done so).
3. Check finger stick glucose value in 10 minutes.
4. If consciousness and cooperation are fully restored and finger stick glucose value is above 80 mg/dL and paramedics have NOT arrived, provide patient with 20 grams of sugar (e.g., 4-5 glucose tablets, 5-6 Lifesavers, 4-6 oz. of a regular [non diet] soft drink, or 8 oz. low fat milk, 4 teaspoons of sugar dissolved in a liquid or juice) and check glucose level again in 10 minutes, and provide with a mixed nutrient drink. Check finger stick glucose value every 10 minutes until paramedics arrive.

HYPERGLYCEMIA

Mild hyperglycemia episodes

For a **hyperglycemic event** to be considered mild, the capillary glucose will exceed 180 mg/dL up to 300 mg/dL as revealed on the CGMS and corroborated by a finger stick reading, the following treatment protocol will be followed:

1. The pump settings have been set up to provide extra amounts of insulin as "boluses" based on target for glycemia and estimated insulin sensitivity index (so-called correction factor) as well as estimated residual insulin from previous boluses (proprietary calculation of Medtronic).

Moderate hyperglycemia episodes

For a hyperglycemia event to be considered moderate, the capillary glucose will exceed 300 mg/dL up to 500 mg/dL as revealed on the CGMS and corroborated by a finger stick reading. The treatment protocol should be followed:

1. If patients are symptomatic or mildly symptomatic, they will be requested to contact the diabetes educator or diabetologist and decisions will be made via the telephone (self adjustment, a visit to the study doctor, emergency department visit) based on individual circumstances.
2. Patients with type 1 diabetes, will be required to test themselves for ketones using the ketone-meters provided to them. If the ketonemia is equal or greater than 0.6 mM/L, the patient will be requested to change the infusion setting of the pump and additional insulin may be provided with injections to avoid further progression into ketoacidosis.
3. If the glycemia and ketonemia worsen, the patient will be requested to visit the emergency department.

Severe hyperglycemia episodes

For a **hyperglycemic event** to be considered severe, the capillary glucose will exceed 500 mg/dL as revealed on the CGMS and corroborated by a finger stick reading. The following treatment protocol will be followed:

1. If patient is symptomatic (polyuria, polydipsia) or with concurrent illness, but capable of good communication, a phone contact to diabetologist is mandatory who will make the decision regarding a visit to see the study doctor or emergency department visit.
2. If symptoms are intense, or the patient is incapable of good communication, immediate transport to emergency department should precede the phone contact.

Safety concerns related to study procedures:

***Gastric Emptying Breath Test*:** Each participant will undergo three ^{13}C-*Spirulina* Gastric Emptying Breath Tests (^{13}C-*Sp*GEBT) in GLUMIT-DG during screening and at 12 and 24 week follow-up visits. The gastric emptying breath test is simple and easily administered. The test should not be administered to participants with a known egg, algae or wheat allergy. Clinical studies were performed validating the ^{13}C-*Sp*GEBT against gastric emptying scintigraphy at the Mayo Clinic, Rochester[15,16]. Furthermore, these studies were conducted to satisfy the FDA's requirement to collect the appropriate efficacy data required for a Pre-Market Approval (PMA) application for commercial distribution. There were no reported adverse events during the development phase of this test.

***EGG with non-caloric and caloric satiety testing*:** Due to the presence of diabetic gastroparesis, meal boluses for the control of postprandial hyperglycemia will be started when the CGMS confirms that glucose concentrations start to rise after Ensure® ingestion. This approach is aimed at minimizing incorrect insulin bolus administration and nutrient due to delayed and possibly erratic

gastric emptying. EGG recording involves placement of EGG electrodes on the abdominal skin. There may be some soreness in removing the EGG electrodes.

Other Risks**:** Blood draw may cause discomfort, such as swelling, temporary sensation of pain, burning, or a bruise that may develop and last for a few days. Less common risks include a blood clot at the site of puncture or swelling of the vein and surrounding tissues, and possible bleeding from the puncture site.

Safety issues related to patient privacy: It is the investigator's responsibility to conduct the protocol under the current version of Declaration of Helsinki, Good Clinical Practice, and rules of local IRBs. The investigator must ensure that the patient's anonymity is maintained in their data submission to the Data Coordinating Center. Patients will be identified only by an identification code but not by their name, SSN, or hospital medical record number. Investigators will maintain a separate confidential enrollment log which matches identifying codes with the patients' names and addresses (i.e., available only to local clinic staff). All study material will be maintained in strict confidence.

6.2. Adherence and retention

Two important goals of the GLUMIT-DG study are to optimize adherence to the glycemic control regimen through use of the CGMS coupled with the insulin pump and to maximize the retention of participants in the study. Assessment of adherence to usage of the CGMS and insulin pump, during the run-in period, will provide clinic staff a means to identify participants having problems with adherence. Adherence will be assessed by:

- Patients displaying the ability to conduct blood glucose monitoring using a standard finger stick method at least 4 times daily
- Patients displaying the ability to successfully wear and manage the CGMS along with the insulin infusion pump evidenced by CGMS usage results
- Patients displaying the ability to electronically transfer CGMS data and insulin infusion pump data to a computer for submission to the study staff

These assessments will guide the consideration of strategies to improve adherence.

6.3. Management of diabetes

All other illnesses will be managed in conjunction with the patient's primary care physician.

Diabetes care will be provided per GLUMIT-DG protocol (see Section 5.5 Follow-up visits and insulin dose adjustments). Treatment approaches for gastroparesis and nutritional counsel are described in the Standard of Care for Patients with Diabetic Gastroparesis (SOP IV) document prepared by the GpCRC Steering Committee.

6.4. Adverse event reporting

The GLUMIT-DG study will monitor and report adverse events to ensure patient safety according to local IRB guidelines and the “Common Rule”, which is a government regulation that requires written procedures and policies for ensuring reporting of “unanticipated problems” involving risks to study participants. The FDA Guidance for Clinical Investigators on Adverse Event Reporting to IRBs - Improving Human Subject Protection[20] document also provides recommendations for adverse event reporting, while specifically focusing on unanticipated event reporting. The FDA recommends that careful review of whether an adverse event is an unanticipated event that must be reported to IRBs should be considered while adhering to local IRB guidelines.

For the GLUMIT-DG study, sentinel events are described as (1) deaths, (2) severe hypoglycemic occurrences, or (3) severe hyperglycemic occurrences. These events will be monitored closely as they may occur during the course of diabetes. While hypoglycemic and hyperglycemic episodes are expected to occur during GLUMIT-DG participation, the frequency of occurrences and the severity of these events will be monitored. Appropriate reporting of severe hypoglycemic and severe hyperglycemic events is outlined throughout this section.

Severe hypoglycemic events occur when a patient’s capillary glucose falls below 50 mg/dL and the patient is incapable of self treatment, hence needing the assistance of a third party for resolution of the hypoglycemia. This type of event will be recorded on study form(s). If a patient has more than two severe hypoglycemic events (i.e., ER visit, relative, etc.) during the study, he/she will be re-evaluated with the diabetologist with an option to be placed back on the previous diabetes medications. Study staff will follow up with patients to ensure that severe hypoglycemic events were properly managed and make any necessary modifications to their treatment.

Severe hyperglycemic events occur when a patient’s capillary glucose is above 500 mg/dL as revealed on the CGMS and corroborated by a finger stick reading. The patient may be symptomatic (polyuria, polydipsia) or with concurrent illness, but capable of good communication; or the patient’s symptoms may be intense, or the patient is incapable of good communication and immediate transport to emergency department may be necessary. These severe hyperglycemic events will be recorded on

study case report forms.

Definitions

Adverse event. An adverse event is any untoward medical occurrence that may present itself during a research study which may or may not have a causal relationship with the treatment or study participation. Adverse events include any unanticipated problems involving risks to participants, or breaches of protocol which might entail risk to participants. The term "unanticipated problem" includes both new risks and increased occurrence of anticipated problems.

Serious adverse event. A serious adverse event (SAE) is an adverse event occurring at any time during the study that results in death, life-threatening adverse experience, inpatient hospitalization or prolongation of existing hospitalization, a persistent or significant disability or incapacity, or a congenital anomaly or birth defect. Severe hypoglycemic and severe hyperglycemic events will be considered an SAE. Other events may also be considered an SAE if, based on medical judgement, the event jeopardized the patient to the point of requiring medical or surgical intervention to prevent the occurrence of any of the conditions for an SAE listed above.

Adverse events will be recorded on case report forms if they are thought to be associated with GLUMIT-DG study participation in accordance with the adverse event definitions. Adverse events may be discovered during regularly scheduled visits or through unscheduled patient contacts between visits. Summary data on adverse events will be reviewed by the GLUMIT-DG Safety Officer and Executive Committee monthly and will be monitored by the GpCRC DSMB quarterly or more frequently, as needed. These summaries will include analyses comparing rates of adverse events by clinic or in other subgroups requested by the DSMB.

After each meeting, the DSMB will issue a written summary of its review of the study data, including adverse events, for transmission to the IRBs at each of the study centers. Analyses or listings of adverse events will not be provided to the IRBs; however, adverse events involving unanticipated problems involving risks to participants, or breaches of protocol which might entail risk to participants will be reported to local IRBs as soon as possible after they are discovered. Each participating center is responsible for ensuring that all local IRB requirements for reporting adverse events are met.

6.5. Reporting and adjudication of serious adverse events

Serious adverse events (SAE) must be reported upon discovery at the clinical center per local

IRB guidelines. This may involve describing the severity of the adverse event and providing further information. The serious adverse event must be reported in the GLUMIT-DG database together with a memo summarizing the circumstances of the event and the current status of the patient. The NIDDK Project Scientist will work with the Data Coordinating Center to transmit the serious adverse events to the DSMB Chair and Steering Committee members and all study investigators.

When clinical centers determine that a serious adverse event (see section 6.4 Adverse event reporting) has occurred, this event must be reported and adjudicated according to the procedures outlined in this section. Examples of SAEs that may occur during the time that a patient is enrolled in the GLUMIT-DG study, that are of particular concern are deaths or severe hypoglycemic or severe hyperglycemic events that may or may not require immediate transport to an emergency department. Any other serious adverse advents that occur will be reported and adjudicated.

Adjudication of each SAE will be done by the standing GLUMIT-DG Serious Adverse Events Adjudication Committee (SAEAC), which has the following membership:

- DCC Safety Officer (L Lee) convenes the GLUMIT-DG SAEAC (Chair)
- Gastroparesis Clinical Research Consortium Chair and Vice-Chair (P Pasricha, H Parkman)
- NIDDK Project Scientist (F Hamilton)
- NIDDK Diabetologist (appointed by F Hamilton)

The SAEAC reviews all pertinent information surrounding the SAE gathered by the DCC from the clinical center and may request additional information, if needed, to determine the etiology of the event. A decision will be made whether a relationship exists between the SAE and participation in the GLUMIT-DG study, as part of the adjudication of the SAE. The study physicians will be removed from the adjudication process, if the SAE under review has occurred at one of their respective clinical centers. In the event that an SAEAC member cannot participate within 5 business days, the NIDDK Project Scientist (Dr. Hamilton) will designate a substitute.

The SAEAC will make one of the following recommendations as a result of its adjudication of the SAE: (1) continue the GLUMIT-DG study without interruption, (2) suspend further enrollment (including any patients that may be in the screening process), (3) stop the study, or (4) any other recommendation that the SAEAC deems appropriate. The SAEAC recommendation, with supporting materials, will be communicated to the DSMB, the Steering Committee, and the NIDDK. Recommendations (2) or (3) will require a Steering Committee conference call to consider the process for suspending or stopping the study, with regard to patient safety. The Steering Committee will develop an action plan and rationale for either resuming or stopping the study or responding to any other recommendation that the SAEAC makes. The action plan and rationale must be reviewed by the DSMB and approved by the NIDDK prior to implementation. All clinical centers must also comply with all reporting requirements for their respective IRBs.

The following specific steps outline the process for reporting and adjudication of an SAE in the GLUMIT-DG study:

1. Within 72 hours of the time that a clinical center determines that an SAE has occurred, the clinical center must 1) notify the DCC by email and telephone that an SAE has occurred, 2) the clinical center must complete, key, and fax the Interim Event form to the DCC and confirm via email or telephone that the form was received, and 3) the clinical center director must complete and send to the DCC a narrative description of the event including the current status of the patient and the planned course for the patient and any other pertinent information.
2. As soon as possible, but no longer than 5 business days from notification of the SAE, the DCC will convene the SAEAC by teleconference to adjudicate the event as outlined above and to make a recommendation to continue, suspend, or stop the GLUMIT-DG study.
3. Within 2 business days, the recommendation and supporting materials will be communicated to the NIDDK, the Steering Committee (i.e., all clinical centers), and to the DSMB.
4. If the recommendation is to either suspend or stop the study, within 1 business day, as a precaution, the DCC will notify all clinical centers to immediately suspend further patient enrollment pending the review of and response to the recommendation.
5 Within 5 business days, the Steering Committee will develop a written plan of action and rationale in response to the recommendation and submit the plan and rationale to the DSMB and to the NIDDK.
6. Within 5 business days, the DSMB will review the action plan or rationale and provide a recommendation to the NIDDK about the action plan and rationale. At the discretion of the DSMB chair and the NIDDK Project Scientist, the DSMB review may be either via

email and email ballot or via a teleconference.

7. Within 3 business days, the NIDDK will communicate its decision regarding any actions that may be required and the DCC and the Steering Committee will move expeditiously to implement these actions.
8. Within 1 month of learning of the SAE, the clinical center will provide an update on the status of the patient to the DCC and details on the resolution of the SAE.
9. The DCC will provide the DSMB with a summary all SAEs as part of the quarterly DSMB reports, including an accounting of adherence to the steps outlined above.

7. Statistical design and analysis

7.1. Hypotheses

Primary hypothesis:

- Treatment with an insulin pump will be safe as evidenced by a limited number and extent of (1) mild, (2) moderate, and (3) severe hypoglycemic events measured by the CGMS

Secondary hypotheses:

- Treatment with an insulin pump will result in improved glycemic control as evidenced by reductions in hemoglobin A1c levels

- Treatment with an insulin pump will result in improvements in clinical manifestations (efficacy measures) of gastroparesis. These will include symptom benefits as measured by a reduction in Gastroparesis Cardinal Symptom Index (GCSI) and Patient Assessment of Upper Gastrointestinal Disorders Quality of Life (PAGI-QOL) scores as well as gastric and autonomic functional benefits including enhancements of gastric emptying, reductions in gastric slow wave tachyarrhythmias, enhanced tolerance of nutrient consumption on satiety testing, and improvements in cardiac autonomic parameters

7.2. Outcome measures

Primary outcome measure:

The primary outcome measure for safety is the weekly frequency of mild, moderate, and severe hypoglycemic events identified by the CGMS. The CGMS and insulin pump data will be uploaded for storage and analysis using the web-based system provided by Medtronic (CareLink Personal http://carelink.minimed.com). The resulting data will be retrieved by the JAEB Center for Health Research for incorporation into a database, which will be transferred to Wake Forest University for derivation of outcome measures and for analysis. The capture and analysis of data will be under the direction of Jorge Calles, MD in collaboration with Boris P. Kovatchev, PhD (University of Virginia Health System, Charlottesville, Virginia).

Along with the absolute numbers, the current CGMS model displays the profile of glycemia in a graphic format and also calculates the percent time in a 24-hour period that the glycemia is within the range considered hypoglycemia, euglycemia or hyperglycemia. For this study, data will be further analyzed using the analytical methods proposed by Kovatchev and colleagues[21]. The CGMS data is assumed to represent accurately the fluctuations in blood glucose that occurs over time and is hence considered in two principal dimensions: risk associated with the amplitude of glucose fluctuations, and time associated with the glucose rates of change. With this understanding, the following parameters will be estimated: temporal glycemia variability (represented by the blood glucose (BG) rate of change).

This parameter estimates the slope of the change for blood glucose as a function of time and is calculated for any given time using the following formula:

[BG(ti) - BG(ti-1)]/(ti - ti-1), where BG = blood glucose and ti and ti-1 are consecutive times.

The data will be averaged over time windows of 1 hour and graphically represented by histograms. The standard deviation of the BG rate of change is a quantitative marker of temporal glycemic variability.

- Low BG Index (LBGI) quantifying the risk associated with both the frequency and the extent of hypoglycemic excursions.
- High BG Index (HBGI) quantifying the risk associated with both the frequency and the extent of hyperglycemic excursions.
- BG risk index = LBGI+HBGI.

These 3 indices are indicators of impending risk of hypoglycemia (LBGI) or marked hyperglycemia (HBGI). The overall BG risk index is an indicator of brittleness in glycemia and indicates risk of both hypo- and hyperglycemia. These 3 indices are computed from CGMS data (with no smoothing) and are typically aggregated in 1 hour blocks. The computation is as follows:

Initially, any given blood glucose is transformed by f(BG) = 1.509 x [(ln(BG))1.084 - 5.381]. The BG risk function is then calculated as r(BG) = 10 x $f(BG)^2$ and separated into left and right branches as follows:

rl(BG) = r(BG) if f(BG) < 0 and 0 otherwise
rh(BG) = r(BG) if f(BG) > 0 and 0 otherwise

For each hour of observation, the LBGI is the average of rl(BG) for a 1-hour period and HBGI is the average of rh(BG) for a 1 hour period. Overall LBGI and HBGI are computed as well as the averages for the entire study period.

Plots of LBGI and HBGI (in time) will be visually analyzed to understand the patient's risk for hypoglycemia and hyperglycemia and reveal times of elevated risks and risk trends. Further analysis of the risk estimates will be performed by Poincaré plots. With this graphic representation, a scattered Poincaré plot indicates system instability (glycemia brittleness) and poor glucose control with high risk of BG fluctuations and hence tendency for hypo- or hyperglycemia. The size of the principal axes of a Poincaré plot is used as a numerical marker of system stability.

Secondary outcome measures:

Effects of insulin pump therapy on the following:

- Hemoglobin A1c at baseline, 12 and 24 week follow-up visits
- Percent time of day in hypoglycemia, euglycemia, or hyperglycemia

- Glycemia variability (blood glucose rate of change)
- Marked hyperglycemia index
- GCSI, PAGI-SYM, and PAGI-QOL scores at baseline, 12, and 24 week follow-up visits
- Gastric emptying rate at 12 and 24 week follow-up visits
- Maximal volume of water and Ensure® consumed during satiety tests at baseline, 12 and 24 week follow-up visits
- Percent tachygastria, bradygastria, and normal 3 cpm activity during EGG testing at baseline, 12 and 24 week follow-up visits
- Vagal cholinergic and sympathetic adrenergic functions at baseline, 12 and 24 week follow-up visits

7.3. Statistical issues

The goals of this pilot study are to assess the safety, feasibility, and potential for efficacy of CGMS in conjunction with insulin pump therapy to produce improved glycemic control in a small sample of diabetic patients with gastroparesis. The findings are intended to provide the foundation for determination of whether a large multicenter randomized, controlled efficacy trial of CGMS in conjunction with insulin pump therapy vs. conventional diabetes management should be conducted in diabetic patients with symptomatic gastroparesis. The current study will provide valuable pilot data to inform the design of an efficacy trial.

As noted above, the primary safety outcome measure for our study is the weekly frequency of mild, moderate and severe hypoglycemic episodes. There is very limited (published or from other sources) information on the frequency of mild, moderate and severe hypoglycemic events in tightly controlled diabetic gastroparesis as measured by CGMS. Thus, for planning purposes, we relied upon a consensus of the clinical experiences of the investigators (Calles, Koch, and Hasler) for estimates needed for the statistical design of this pilot study:

- Assumed mean weekly mild to moderate hypoglycemic episodes by CGMS: 0.6
- Assumed SD for weekly mild to moderate hypoglycemic episodes by CGMS: 0.8
- Assumed intra-class correlation between baseline and on-treatment weekly mild, moderate and severe hypoglycemic episodes by CGMS: 0.6
- Estimated variance of change from baseline (a minimum of 4 weeks up to 16 weeks) to on-treatment (24 weeks) mean weekly mild, moderate and severe hypoglycemic episodes (estimated from SD and intraclass correlation, accounting for 3:1 ratio of on-treatment to baseline period .

We planned the study to test the safety hypothesis that, compared to a minimum of 4 weeks up to 16 weeks of baseline treatment with CGMS, any increase in mean weekly mild, moderate and severe hypoglycemic events during approximately 24 weeks of insulin pump treatment and CGMS was no larger than 35%. Using a one-sample equivalence z-test and assuming one-sided Type I error of 0.05, power =0.9, expected percent increase from baseline to on-treatment=0%, and equivalence limit of 35% (0.6 vs. 0.81 episodes per week), the calculated sample size was N=37. The method of

calculation and software used are as follows:

- Method for sample size calculation: One sample test for equivalence (Chow, Shao and Wang, Sample Size Calculations In Clinical Research, Taylor & Francis, NY, 2003: p.52-53)[22]
- Software: Centre for Clinical Trials, Chinese University of Hong Kong[23] http://www.cct.cuhk.edu.hk/stat/mean/osm_equivalence.htm

We increased the target sample size approximately 10% to N=40 patients to allow departures from assumptions. In addition, we plan to review safety data with the DSMB and assumptions used for the sample size calculation when approximately N=10 patients have safety data and have completed the study to re-assess the adequacy of the total sample size.

An important, but secondary, efficacy outcome is reduction in hemoglobin A1c. However, if we assume that 25% (10 patients) do not achieve good glycemic control (hemoglobin A1c >7% at 12 and 24-weeks), the confidence limits on the actual proportion not achieving good control with 40 patients will be wide with a half-width of ±16%. However, a sample size of 40 will provide adequate power to detect clinically meaningful changes in measures of gastroparesis symptoms, quality of life, or on measures of gastric motor and sensory and autonomic function. For example, a 25% reduction in mean from a baseline GCSI score of 27±15 to 20±15, with a sample size of 40, can be detected with >99% power, again assuming a Type I error of 0.05 and a modest intra-class correlation between baseline and follow-up GCSI measures. We will compare Type 1 versus Type 2 diabetics for indications of differential outcomes, however, the power for such comparisons is much less -- only 57% power for the hypoglycemic outcome measure with equal numbers of Type 1 and Type 2 diabetics and less power if the numbers are unequal. These efficacy and subgroup comparisons for differential responses are best addressed in the context of a larger multicenter, randomized, controlled trial.

7.4. Interim analysis

An independent Data and Safety Monitoring Board (DSMB), appointed by the NIDDK, is responsible for approving the protocol for the GLUMIT-DG study and for monitoring the accumulated interim data as the study progresses to ensure patient safety and to review efficacy. The DSMB is a multi-disciplinary group with a written charge provided by the NIDDK. The DSMB reports to the NIDDK, which will, in turn, communicate DSMB recommendations to the investigators, as appropriate.

The DSMB meets to approve the protocol. After the study commences, the DSMB meets twice a year to review data or other performance issues. The DSMB may request more frequent meetings if necessary to fulfill its charge. It may also request additional safety reports on a more frequent basis. For example, all serious adverse events are reported to the DSMB quarterly for their consideration and recommendations as they occur.

Interim data on all safety measures requested by the DSMB are reviewed at each of the scheduled semi-annual full meetings. Two additional written safety reports will be reviewed by the DSMB between scheduled full meetings. Serious adverse events will be reviewed by the DSMB quarterly as they occur with the option of a teleconference discussion if any DSMB member so requests.

No formal stopping guidelines are proposed for this pilot study of safety. However, each DSMB periodic monitoring report will include an analysis of the primary safety outcome that calculates, based on the available data, the lower 95% confidence limit on the estimated percent increase from baseline in mean weekly mild to moderate or severe hypoglycemic events during the on-treatment period. If the lower confidence limit is greater than 35%, the DSMB will be asked to discuss and vote on whether the study should continue, based on a review of all information available at that time.

The DSMB also reviews the overall progress of the study in terms of recruitment and data quality and makes a formal recommendation to the NIDDK at the end of each scheduled meeting as to whether the study should continue unmodified, continue with protocol modifications, or be stopped.

8. Human subjects issues

Overview and IRB approval

The study protocol and consent form will be submitted to each clinical center's IRB. Additionally, each clinical center will submit to their IRB any recruitment materials to be used at their site. A clinical center may not initiate any patient contact about the GLUMIT-DG study until the center has IRB approval and the DCC has certified the center for initiation of patient activities. Consent form must have IRB approval. Centers must provide the DCC with copies of the initial IRB approval notice and subsequent renewals, as well as copies of the IRB approved consent statement. All study personnel must have completed training in the Protection of Human Subjects, per NIH guidelines.

The proposed study anticipates recruiting a significant proportion of women and racial/ethnic minorities (African American, Hispanics or Latino, Asian, American Indian/Alaska Native, Native Hawaiian or other Pacific Islander) as well as non-Hispanic white subjects. We anticipate that the patients will be recruited from diverse sources, largely from tertiary referral populations and will represent the entire spectrum of diabetic gastroparesis.

All patients enrolled in the GLUMIT-DG study will receive the standard of care for diabetic gastroparesis and associated medical problems as defined by the GpCRC Steering Committee (see Standard of Care for Patients with Diabetic Gastroparesis). This will include provision of health care counseling and educational materials at enrollment and during follow-up.

Informed Consent

A prototype consent will be prepared for the study. Individual centers may add material but may not delete material thought to be necessary for informed consent. Centers may reformat and reword information to conform to their local IRB requirements. A signed consent form will be obtained from the subject. The consent form will describe the purpose of the study, the procedures to be followed, and the risks and benefits of participation. A copy of the consent form will be given to the subject and this fact will be documented in the subject's record.

Confidentiality

All laboratory specimens, evaluation forms, reports, and other records that are part of the study data collection and entry materials will be identified by coded number only to maintain subject confidentiality. All records will be kept in locked file cabinets with access limited to GpCRC investigators. All computer entry and networking programs will identify patients by patient identification number. Clinical information will not be released without written permission of the subject, except as necessary for monitoring by the IRB or DSMB. Clinical information may be reviewed during site visits, but use of personal identifiers will be avoided. Consent procedures and forms, and the communication, transmission and storage of patient data will comply with individual center IRB and NIH requirements for compliance with the Health Insurance Portability and Accountability Act (HIPAA).

9. References

1. Soykan I, Sivri B, Sarosiek I, Kiernan B, McCallum RW. Demography, clinical characteristics, psychological and abuse profiles, treatment, and long-term follow-up of patients with gastroparesis. Dig Dis Sci 1998;43:2398-2404.

2. The Diabetes Control and Complications Trial Research Group. The effect of intensive therapy of diabetes on the development and progression of long-term complications in insulin-dependent diabetes mellitus. N Engl J Med 1993;329:977-986.

3. Bytzer P, Talley NJ, Leemon M, Young, LJ, Jones, MP, Horowitz. Prevalence of gastrointestinal symptoms associated with diabetes mellitus. Arch Intern Med 2001;161:1989-1996.

4. Soykan, I, Sivri B, Saroseik I, Kiernan, B, McCallum, RW. Demography, clinical characteristics, psychological and abuse profiles, treatment, and long-term follow-up of patients with gastroparesis. Dig Dis Sci 1998;43:2398-2404.

5. Hoogerwerf WA, Pasricha PJ, Kalloo AN, Schuster MM. Pain: the overlooked symptom in gastroparesis. Am J Gastroenterol 1999;94:1029-1033.

6. Horowitz M, Maddox AF, Wishart JM, Harding, PE, Chatterton, BE, Shearman, DJ. Relationships between oesophageal transit and solid and liquid gastric emptying in diabetes mellitus. Eur J Nuc Med 1991;18: 229-234.

7. Horowitz M, Harding PE, Maddox AF, Wishart, JM, Akkermans, LM, Chatterton, BE, Shearman, DJ. Gastric and esophageal emptying in patients with type 2 (non-insulin-dependent) diabetes mellitus. Diabetologia 1989;32:151-159.

8. Schvarcz E, Palmer M, Aman J, Lindkvist, V, Beckman, KW. Hypoglycaemia increases the gastric emptying rate in patients with type I diabetes mellitus. Diabetes Med 1993;10:660-663.

9. Jebbink RJ, Samsom M, Bruijs PP, Bravenboer, B, Akkermans, LM, Vanberge-Henegouwen, GP, Smout, AJ. Hyperglycemia induces abnormalities of gastric myoelectrical activity in patients with type I diabetes mellitus. Gastroenterology 1994;107: 1390-1397.

10. Hasler WL, Soudah HC, Dulai G, Owyang C. Mediation of hyperglycemia-evoked gastric slow wave dysrhythmias by endogenous prostaglandins. Gastroenterology 1995;108:727-736.

11. Samsom M, Salet GA, Roelofs JM, Akkermans, LM, Vanberge-Henegouwen, GP, Samout, AJ. Compliance of the proximal stomach and dyspeptic symptoms in patients with type I diabetes mellitus. Dig Dis Sci 1995;40:2037-2042.

12. Weber KK, Lohmann T, Busch K, Donati-Hirsch, I, Riel, R.. High frequency of unrecognized hypoglycaemias in patients with Type 2 diabetes is discovered by continuous glucose monitoring. Exp Clin Endocrinol Diab 2007;115:491-494.

GLUMIT-DG Protocol

13. Bailey TS, Zisser HC, Garg SK. Reduction in hemoglobin A1C with real-time continuous glucose monitoring: results from a 12-week observational study. Diab Technol Ther 2007;9: 203-210.

14. Wentholt IM, Maran A, Masurel N, Heine, RJ, Hoekstra, JB, DeVriese, JH. Nocturnal hypoglycaemia in Type 1 diabetic patients, assessed with continuous glucose monitoring: frequency, duration and associations. Diab Med 2007;24:527-532.

15. Viramontes BE, Kim DY, Camilleri M, Lee JS, Stephens D, Burton DD, Thomforde GM, Klein PD, Zinsmeister AR. Validation of a stable isotope gastric emptying test for normal, accelerated or delayed gastric emptying. Neurogastroenterol Motil 2001;13:567-74.

16. Lawrence SA, Camilleri M, Vella A, Burton D, Baxter K, Simonson J, Zinsmeister, AR. A stable isotope breath test with a standard meal for abnormal gastric emptying of solids in the clinic and in research. Clin Gastroenterol Hepatol 2008;6:635-643.

17. Rentz AM, Kahrilas P, Stanghellini, Tack J, Talley NJ, de la Loge C, Trudeau E, Dubois D, Revicki DA. Development and psychometric evaluation of the patient assessment of upper gastrointestinal symptom severity index (PAGI-SYM) in patients with upper gastrointestinal disorders. Qual Life Res 2004;13:1737-1749.

18. de la Lorge C, Trudeau E, Marquis P, Kahrilas P, Stnaghellini V, Talley NJ, Tack J, Revicki DA, Rentz AM, Dubois D. Cross cultural development and validation of a patient self-administered questionnaire to access quality of life in upper gastrointestinal disorders: the PAGI-QOL. Qual Life Res 2004:13:1751-1762.

19. Common Terminology Criteria for Adverse Events v3.0, publication date August 9, 2006. Accessed at http://ctep.cancer.gov/reporting/ctc.html on 4 April 2007.

20. Guidance for Clinical Investigators, Sponsors, and IRBs Adverse Event Reporting to IRBs - Improving Human Subject Protection, publication date January 2009. Accessed at http://www.fda.gov/cder/guidance/oc2008150.fnl.htm.

21. Kovatchev BP, Clarke WL, Breton M, Braymank, McCall A. Quantifying temporal glucose variability in diabetes via continuous glucose monitoring: mathematical methods and clinical application. Diabetes Technol Ther 2005;849-862.

22. Chow SC, Shao J, Wong, H. Sample size calculations in clinical research. Taylor & Francis, New York, 2003: 52-53.

23. Center for Clinical Trials Software, Chinese University of Hong Kong. Accessed at http://www.cct.cubk.edu.hk/stat/mean/osm_equivalence.htm.

10. Appendices

10.1. Participating centers 47
10.2. Data collection schedule 48
10.3. Whole blood draw schedule 49
10.4. Glossary 50

10.1. Participating centers

Clinical Centers

- Stanford University
  - California Pacific Medical Center
- Temple University
- Texas Tech University
- University of Michigan
- University of Mississippi Medical Center
- Wake Forest University Medical Sciences

Data Coordinating Center:

- Johns Hopkins University

National Institutes of Health:

- National Institute of Diabetes and Digestive and Kidney Diseases

---

10.2. Data collection schedule

	Screening, run-in, and enrollment visits				Follow-up visits Weeks from enrollment					
**Assessment/Procedure**	**s1**	**s2**	**s3**	**R***	**4**	**8**	**12**	**16**	**20**	**24**
Consent	X	.	.	.	.	.	.	.	.	.
Gastric emptying scintigraphy review	X	.	.	.	.	.	.	.	.	.
Upper endoscopy results review	X	.	.	.	.	.	.	.	.	.
Baseline medical history	X	.	.	.	.	.	.	.	.	.
Follow-up medical history	.	.	.	.	X	X	X	X	X	X
Physical exam	X	.	.	.	.	.	X	.	.	X
Autonomic function and ECG	.	X	.	.	.	.	X	.	.	X
PAGI-QOL questionnaire	X	.	.	.	.	.	X	.	.	X
PAGI-SYM questionnaire	X	.	.	.	.	.	X	.	.	X
Gastric emptying breath test (GEBT)	.	X	.	.	.	.	X	.	.	X
Water load and satiety test with	.	.	X	.	.	.	X	.	.	X
CGMS training	X	X	X	.	.	.	.	.	.	.
Insulin pump training with CGMS	.	.	X	X	.	.	.	.	.	.
Adverse event monitoring	.	X	X	X	X	X	X	X	X	X
CBC, HbA1c, metabolic panel	X	.	.	.	.	.	X	.	.	X
Thyroid stimulating hormone	X	.	.	.	.	.	.	.	.	.
Plasma banking	.	X	.	.	.	.	X	.	.	X

*__Run-in__: Four visits include training with the insulin pump and CGMS to establish the baseline parameters.
Physical exam includes measurement of weight, vital signs (temperature, heart rate, blood pressure), general physical findings
Complete blood count (CBC): white blood cells, red blood cells, white blood cell differential, hemoglobin, hematocrit, platelets
HbA1c: Hemoglobin A1c
Metabolic panel: sodium, potassium, chloride, carbon dioxide, glucose, ketonemia, calcium, blood urea nitrogen (BUN) creatinine, albumin, total protein, alanine aminotransferase (ALT), aspartate aminotransferase (AST), bilirubin
*Follow-up visits at weeks 12 and 24 require two days of testing: day 1 is for gastric emptying breath test and day 2 for electrogastrogram with water load and satiety testing.

10.3. Whole blood draw schedule

**Procedure**	**Study visit (wk)**							
	**Screening**	**4**	**8**	**12**	**16**	**20**	**24**	**Total**
Hemoglobin A1c	**5**	**.**	**.**	**5**	**.**	**.**	**5**	**15**
Complete blood count	**5**	**.**	**.**	**5**	**.**	**.**	**5**	**15**
Metabolic panel	**5**	**.**	**.**	**5**	**.**	**.**	**5**	**15**
Thyroid stimulating hormone	**5**	**.**	**.**		**.**	**.**		**5**
Blood for plasma banking	**10**	**.**	**.**	**10**	**.**	**.**	**10**	**30**
**Total (in mL)**	**30**	**.**	**.**	**25**	**.**	**.**	**25**	**80**

Complete blood count: white blood cells, red blood cells, white blood cell differential, hemoglobin, hematocrit, platelets
Metabolic panel: sodium, potassium, chloride, carbon dioxide, glucose, calcium, blood urea nitrogen (BUN) creatinine, albumin, total protein, alanine aminotransferase (ALT), aspartate aminotransferase (AST), bilirubin

10.4. Glossary

BG	-	blood glucose
BMI	-	body mass index (kg/m^2)
BW	-	body weight
CBC	-	complete blood count
CGMS	-	continuous glucose monitoring system
CHO	-	carbohydrate
CHO:I	-	carbohydrate to insulin ratio
CMP	-	complete metabolic panel
CTCAE	-	Common Terminology Criteria for Adverse Events
DCC	-	Data Coordinating Center
DSMB	-	Data and Safety Monitoring Board
EGG	-	electrogastrography
ECG	-	electrocardiogram
FDA	-	Food and Drug Administration
GEBT	-	gastric emptying breath testing
GCSI	-	Gastroparesis Cardinal Symptom Index
GLUMIT-DG	-	Pilot Study of the Safety, Feasibility, and Potential Efficacy of Continuous Glucose Monitoring and Insulin Pump Therapy in Diabetic Gastroparesis
GpCRC	-	Gastroparesis Clinical Research Consortium
GpR	-	Gastroparesis Registry
HBGI	-	high blood glucose index
HbA1c	-	glycosylated hemoglobin A1c
HIPAA	-	Health Insurance Portability and Accountability Act
IRB	-	Institutional Review Board
LBGI	-	low blood glucose index
NIDDK	-	National Institute of Diabetes and Digestive and Kidney Diseases
PAGI-QOL	-	Patient Assessment of Upper Gastrointestinal Disorders - Quality of Life
PAGI-SYM	-	Patient Assessment of Upper Gastrointestinal Disorders - Symptom
SAE	-	serious adverse event
SAEAC	-	Serious Adverse Events Adjudication Committee
SOP	-	standard operating procedures
TSH	-	thyroid stimulating hormone
