# Supplementary material for: Glucose sensor-augmented continuous subcutaneous insulin infusion in patients with diabetic gastroparesis: An open-label pilot prospective study
Source: PLoS One. 2018 Apr 13;13(4):e0194759. doi: 10.1371/journal.pone.0194759 (PMC5898706; doi:10.1371/journal.pone.0194759)
Supplement: S1 Tables — This file includes Tables A, B, C, D, E, and F. (PDF) [file pone.0194759.s006.pdf]

Supplemental Tables

Table A. Current Type of Insulin, Insulin Pump and Antidiabetic Agents in Past Month at Screening Visit.

Type of Diabetes Therapy	Specific Products	All Patients with Diabetes (N=45)	T1DM Patients (N=32)	T2DM Patients (N=13)	P-value (T1DM vs. T2DM)†
Current type of insulin					0.27
	Lantus	1	0	1	
	Lantus + Novolog	6	4	2	
	Lantus + Novolin	1	1	0	
	Lantus + Humalog	8	3	5	
	Lantus + Humalog + Novolog	1	0	1	
	Lantus + Other*	1	1	0	
	Novolog	10	8	2	
	Humalog	12	10	2	
	Humalog + Humulin	1	1	0	
	Levemir + Novolog	1	1	0	
	Levemir + Humalog	2	2	0	
	Apridra	1	1	0	
Current type of insulin pump					0.28
	None	23	13	10	
	Minimed Paradigm REAL-Time	18	15	3	
	One Touch Ping	2	2	0	
	Cosmo	1	1	0	

	Rebel Medtronics	1	1	0	
Type of other antidiabetic agent					
	Amaryl	1	1	0	1.00
	Micronase	1	1	0	1.00
	Glucophage	6	2	4	0.05
	Actos	2	1	1	0.50
	Januvia	2	1	1	0.50

* 70/30 (insulin not specified)

† Based on Fisher's exact test

Table B. Other Symptom and Quality of Life Characteristics at Screening.

Category	Variable	All Patients With Diabetes (N=45)	T1DM Patients (N=32)	T2DM Patients (N=13)	P Value (T1DM vs. T2DM)*
Gastroparesis symptoms					
	Nausea component	3.5 (1.2)	3.4 (1.2)	3.6 (1.3)	0.67
	Retching component	2.4 (1.6)	2.2 (1.6)	2.6 (1.6)	0.49
	Vomiting component	2.3 (1.9)	2.2 (1.9)	2.4 (1.9)	0.80
	Stomach fullness component	3.9 (0.9)	3.8 (1.0)	4.0 (0.7)	0.56
	Not able to finish meal component	3.3 (1.3)	3.4 (1.3)	3.2 (1.4)	0.75
	Feeling excessively full component	3.9 (1.0)	3.9 (1.1)	3.8 (0.9)	0.67
	Loss of appetite component	3.0 (1.6)	2.9 (1.6)	3.8 (1.5)	0.35
	Bloating component	3.7 (1.2)	3.6 (1.3)	4.2 (0.8)	0.08
	Stomach visibly larger component	3.4 (1.4)	3.3 (1.4)	3.5 (1.6)	0.61
Quality of life					
	Daily activities component	2.3 (1.2)	2.6 (1.1)	1.8 (2.2)	0.05
	Clothing component	2.5 (1.7)	2.7 (1.7)	2.1 (1.5)	0.23
	Diet and food habits component	1.7 (1.3)	1.8 (1.2)	1.3 (1.3)	0.27
	Relationship component	2.9 (1.5)	3.1 (1.5)	2.5 (1.5)	0.26

	Psychological well-being and distress component	2.3 (1.3)	2.7 (1.3)	1.6 (1.3)	0.02

All values are either N (%) or mean (SD).

GCSI individual scores are coded from 0=none to 5=very severe.

PAGI-QOL domains are coded from 0=lowest quality of life to 5=highest quality of life.

* Based on Fisher's exact test for categorical variables and unequal variance t-test for means.

Table C. Factors Associated with Serious Adverse Events.

Category	Variable	Patients Without SAEs N=37	Patients With SAEs N=8	P Value*
Demographic/clinical				
	Female	27 (73%)	4 (50%)	0.23
	Age (yr)	45 (12)	44 (12)	0.84
	White	30 (81%)	7 (88%)	1.00
	Hispanic	8 (22%)	3 (38%)	0.38
	Diabetes duration (yr)	20 (12)	21 (11)	0.85
	Body mass index (kg/m^2)	29 (8)	28 (7)	0.61
Medication use				
	Antidiabetic medications (other than insulin)	6 (16%)	0 (0%)	0.57
	Proton pump inhibitors	27 (73%)	5 (62%)	0.67
	Prokinetics	17 (46%)	4 (50%)	1.00
	Antiemetics	17 (46%)	7 (88%)	0.05
	Tricyclic antidepressants	7 (19%)	2 (25%)	0.65
Metabolic				
	Type 1 diabetes	25 (68%)	7 (88%)	0.40
	Hemoglobin A1c (%)	9.4 (1.4)	9.0 (1.0)	0.33
Gastric emptying				
	2 hr gastric retention (%)	64 (19)	62 (23)	0.82
	4 hr gastric retention (%)	31 (20)	37 (22)	0.52
Gastroparesis symptoms				
	Total GCSI score	28.0 (6.8)	35.6 (5.4)	0.004

	Total nausea/vomiting subscore	7.3 (4.0)	11.9 (2.9)	0.002
	Nausea component	3.3 (1.2)	4.5 (0.8)	0.002
	Retching component	2.0 (1.5)	3.9 (1.0)	0.0006
	Vomiting component	2.0 (1.8)	3.5 (1.9)	0.07
	Total fullness/early satiety subscore	13.7 (3.7)	16.2 (2.8)	0.04
	Stomach fullness component	3.8 (0.9)	4.1 (0.8)	0.41
	Not able to finish meal component	3.1 (1.3)	4.2 (0.9)	0.01
	Feeling excessively full component	3.8 (1.0)	4.4 (0.7)	0.07
	Loss of appetite component	2.9 (1.6)	3.5 (1.2)	0.29
	Total bloating/distention subscore	7.0 (2.3)	7.5 (2.4)	0.61
	Bloating component	3.8 (1.2)	3.6 (1.3)	0.80
	Stomach visibly larger component	3.2 (1.4)	3.9 (1.6)	0.34
Quality of life				
	Mean PAGI-QOL score	2.4 (1.1)	2.1 (1.5)	0.60
	Daily activities component	2.5 (1.2)	1.7 (0.9)	0.05
	Clothing component	2.4 (1.7)	3.4 (1.5)	0.11
	Diet and food habits component	1.7 (1.3)	1.5 (1.0)	0.70
	Relationship component	3.1 (1.4)	2.1 (1.8)	0.18
	Psychological well-being and distress component	2.4 (1.3)	2.1 (1.5)	0.65
Satiety testing				
	Water load (mL)	419 (193)	484 (276)	0.57
	Liquid nutrient (mL)	409 (271)	473 (184)	0.46

All values are either N (%) or mean (SD).

* Based on Fisher's exact test for categorical variables and unequal variance t-test for means.

Table D. Effect of CSII Plus CGM Treatment on Individual Gastroparesis Symptoms and Quality of Life Domains.

Category	Variable	Screening Score Mean (SD)	12 Weeks Treatment			24 Weeks Treatment		
			Treatment Score Mean (SD)	Difference Treatment Minus Screening Mean (SD)	P Value*	Treatment Score Mean (SD)	Difference Treatment Minus Screening Mean (SD)	P Value*
Symptoms								
	Nausea component	3.5 (1.2)	2.4 (1.5)	-1.0 (-1.5, -0.5)	0.0001	2.4 (1.5)	-1.0 (-1.5, -0.5)	<0.0001
	Retching component	2.4 (1.6)	1.2 (1.6)	-1.0 (-1.5, -0.5)	0.0002	1.3 (1.6)	-1.0 (-1.5, -0.5)	0.0001
	Vomiting component	2.3 (1.9)	1.3 (1.6)	-0.8 (-1.4, -0.3)	0.003	1.3 (1.6)	-0.9 (-1.4, -0.3)	0.003
	Stomach fullness component	3.9 (0.9)	3.0 (1.1)	-0.9 (-1.3, -0.6)	<0.0001	3.1 (1.3)	-0.7 (-1.1, -0.3)	0.0006
	Not able to finish meal component	3.3 (1.3)	2.5 (1.4)	-0.8 (-1.3, -0.3)	0.004	2.6 (1.3)	-0.6 (-1.2, 0.0)	0.05
	Feeling excessively full component	3.9 (1.0)	3.1 (1.3)	-0.7 (-1.2, -0.3)	0.003	3.1 (1.4)	-0.7 (-1.2, -0.3)	0.001
	Loss of appetite component	3.0 (1.6)	2.4 (1.5)	-0.6 (-1.1, -0.1)	0.01	2.5 (1.5)	-0.6 (-1.0, -0.1)	0.01
	Bloating component	3.7 (1.2)	3.0 (1.4)	-0.8 (-1.1, -0.5)	<0.0001	2.9 (1.5)	-0.8 (-1.2, -0.5)	<0.0001
	Stomach visibly larger component	3.4 (1.4)	2.9 (1.5)	-0.5 (-1.0, 0.0)	0.04	2.6 (1.7)	-0.7 (-1.2, -0.3)	0.003
Quality of life								
	Daily activities component	2.3 (1.2)	2.9 (1.2)	0.5 (0.2, 0.8)	0.002	2.8 (1.3)	0.4 (0.1, 0.8)	0.02
	Clothing component	2.5 (1.7)	3.0 (1.6)	0.5 (0.1, 0.9)	0.02	3.2 (1.5)	0.6 (0.2, 1.1)	0.003

	Diet and food habits component	1.7 (1.3)	2.4 (1.3)	0.7 (0.3, 1.0)	0.0008	2.4 (1.5)	0.7 (0.3, 1.1)	0.0006
	Relationship component	2.9 (1.5)	3.8 (1.4)	0.8 (0.4, 1.3)	0.0003	3.8 (1.2)	0.8 (0.5, 1.2)	<0.0001
	Psychological well-being and distress component	2.3 (1.3)	3.1 (1.3)	0.8 (0.4, 1.2)	0.0003	3.0 (1.3)	0.7 (0.3, 1.1)	0.002

All values are either N (%) or mean (SD).

* Based on paired t-test of mean change=0.

Table E. Adverse Events During Screening and Treatment Phases by Diabetes Subtype.

Parameter		T1DM				T2DM			
		**Screening/ Run-In Phase* (N=32)**	**Intervention Phase (N=29)**	**Relative Risk (95% CI) Screening/ Run-in vs. Treatment**	**P Value †**	**Screening/ Run-In Phase* (N=13)**	**Intervention Phase (N=13)**	**Relative Risk (95% CI) Screening/ Run-in vs. Treatment**	**P Value †**
Mild, moderate, or severe hypoglycemic events									
	Number of events	478	1162			116	442		
	Number of patients with event	30	26			13	10		
	Rate per person-week	2.2	2.2	1.0 (0.7, 1.4)	0.95	1.0	2.1	2.0 (1.1, 3.3)	0.03
Severe hypoglycemic events									
	Number of events	1	5			0	1		
	Number of patients with event	1	5			0	1		

	Rate per person-year	0.14	0.36	2.5 (0.4, 10.0)	0.31	0.00	0.16	N/C	NA
Gastroparesis exacerbations ‡									
	Number of events	7	9			2	2		
	Number of patients with event	3	5			1	2		
	Rate per person-year	1.00	0.64	0.5 (0.1, 2.5)	0.42	0.62	0.32	0.5 (0.1, 2.0)	0.36
Other¶									
	Number of events	2	5			2	1		
	Number of patients with event	2	4			1	1		
	Rate per person-year	0.29	0.36	1.2 (0.2, 10.0)	0.80	0.62	0.16	0.3 (0.02, 3.3)	0.28

* Includes Run-In phase.

† Poisson regression using generalized estimating equations to account for within patient correlation across study phases.

‡ Includes nausea, vomiting, abdominal pain, diarrhea.

¶ Cholecystectomy, bilateral otitis media (2), hyperglycemia (3), rash, dizziness (2), retinal detachment.

Table F. Effect of CSII Plus CGM Treatment on Glycemic Control, Symptoms, Quality of Life, and Meal Tolerance by Diabetes Subtype.

Category	Variable	T1DM Patients			T2DM Patients			T1DM Minus T2DM Mean (95% CI)	P Value†
		Screening	24 Week Treatment	P Value†	Screening	24 Week Treatment	P Value†		
Metabolic									
	Hemoglobin A1c (%)	9.4 (1.3)	8.8 (1.2)	0.002	9.3 (1.6)	7.4 (1.2)	0.01	1.3 (0.5, 2.1)	0.002
Symptoms									
	Total GCSI score	28.8 (7.0)	19.1 (9.6)	<0.0001	30.7 (7.6)	28.0 (8.9)	0.19	-7.3 (-13.0, -1.5)	0.01
	Total nausea/vomiting subscore	7.9 (4.1)	4.2 (4.0)	0.0003	8.6 (4.4)	6.9 (4.6)	0.05	-2.2 (-4.7, 0.3)	0.08
	Nausea component	3.4 (1.2)	2.0 (1.5)	0.0002	3.6 (1.3)	3.3 (1.2)	0.22	-1.2 (-2.1, -0.3)	0.01
	Retching component	2.2 (1.6)	1.1 (1.4)	0.001	2.6 (1.6)	1.7 (1.9)	0.04	-0.3 (-1.3, 0.6)	0.46
	Vomiting component	2.2 (1.9)	1.1 (1.5)	0.008	2.4 (1.9)	1.9 (1.8)	0.19	-0.7 (-0.7, 0.2)	0.13
	Total fullness/early satiety subscore	14.0 (3.8)	10.2 (4.6)	0.0005	14.4 (3.3)	13.8 (4.0)	0.57	-3.2 (-6.1, -0.4)	0.03
	Stomach fullness component	3.8 (1.0)	2.8 (1.3)	0.0002	4.0 (0.7)	3.9 (1.0)	0.78	-1.0 (-1.8, -0.2)	0.01
	Not able to finish meal component	3.4 (1.3)	2.4 (1.3)	0.02	3.2 (1.4)	3.2 (1.2)	1.00	-0.9 (-1.7, 0.0)	0.06
	Feeling excessively full component	3.9 (1.1)	2.8 (1.5)	0.0004	3.8 (0.9)	3.8 (1.1)	1.00	-1.0 (-1.9, -0.2)	0.02

	Loss of appetite component	2.9 (1.6)	2.3 (1.5)	0.06	3.4 (1.5)	2.8 (1.5)	0.07	-0.3 (-1.1, 0.6)	0.53
	Total bloating/ distention subscore	6.8 (2.3)	4.7 (2.8)	<0.0001	7.7 (2.3)	7.3 (2.8)	0.57	-2.0 (-3.5, -0.4)	0.02
	Bloating component	3.6 (1.3)	2.5 (1.5)	<0.0001	4.2 (0.8)	3.8 (1.3)	0.37	-0.9 (-1.7, -0.1)	0.02
	Stomach visibly larger component	3.3 (1.4)	2.2 (1.6)	0.001	3.5 (1.6)	3.5 (1.7)	0.84	-1.0 (-2.0, -0.1)	0.03
Quality of life									
	Mean PAGI-QOL score	2.6 (1.1)	3.3 (1.1)	0.002	1.8 (1.1)	2.6 (0.7)	0.16	0.2 (-0.4, 0.9)	0.43
	Daily activities component	2.6 (1.1)	3.1 (1.3)	0.03	1.8 (1.2)	2.3 (1.1)	0.01	0.3 (-0.5, 1.0)	0.48
	Clothing component	2.7 (1.7)	3.3 (1.4)	0.08	2.1 (1.5)	2.8 (1.6)	0.04	0.1 (-0.7, 0.9)	0.81
	Diet and food habits component	1.8 (1.2)	2.8 (1.6)	0.11	1.3 (1.3)	1.7 (1.1)	0.09	0.6 (-0.2, 1.5)	0.13
	Relationship component	3.1 (1.5)	3.9 (1.3)	0.02	2.5 (1.5)	3.6 (1.0)	0.06	0.0 (-0.6, 0.7)	0.89
	Psychological well-being and distress component	2.7 (1.3)	3.2 (1.3)	0.02	1.6 (1.3)	2.6 (1.1)	0.008	0.1 (-0.8, 0.9)	0.88
Satiety testing									
	Water load (mL)	476 (208)	491 (239)	0.91	326 (168)	267 (158)	0.12	123 (-14, 260)	0.08
	Liquid nutrient (mL)	470 (263)	570 (333)	0.04	293 (202)	321 (184)	0.91	91 (-40, 222)	0.17

All values are either N (%) or mean (SD).

Paired t-test of comparison of screening and 24 week treatment value within diabetes subtype.

† Based on ANCOVA model regressing change on indicator variable of diabetes subtype and baseline value of outcome.
